# The impact of skin care products on skin chemistry and microbiome dynamics

**DOI:** 10.1186/s12915-019-0660-6

**Published:** 2019-06-12

**Authors:** Amina Bouslimani, Ricardo da Silva, Tomasz Kosciolek, Stefan Janssen, Chris Callewaert, Amnon Amir, Kathleen Dorrestein, Alexey V. Melnik, Livia S. Zaramela, Ji-Nu Kim, Gregory Humphrey, Tara Schwartz, Karenina Sanders, Caitriona Brennan, Tal Luzzatto-Knaan, Gail Ackermann, Daniel McDonald, Karsten Zengler, Rob Knight, Pieter C. Dorrestein

**Affiliations:** 1 Collaborative Mass Spectrometry Innovation Center, Skaggs School of Pharmacy and Pharmaceutical Sciences, San Diego, USA; 2 Department of Pediatrics, University of California, San Diego, La Jolla, CA 92037 USA; 3 Department for Pediatric Oncology, Hematology and Clinical Immunology, University Children’s Hospital, Medical Faculty, Heinrich-Heine-University Düsseldorf, Düsseldorf, Germany; 4 Center for Microbial Ecology and Technology, Ghent University, 9000 Ghent, Belgium; 5 Center for Microbiome Innovation, University of California, San Diego, La Jolla, CA 92307 USA; 6 Department of Bioengineering, University of California, San Diego, La Jolla, CA 92093 USA; 7 Department of Computer Science and Engineering, University of California, San Diego, La Jolla, CA 92093 USA; 8 Department of Pharmacology, University of California, San Diego, La Jolla, CA 92037 USA

**Keywords:** Skin, Skin care products, Mass spectrometry, Metabolomics, 16S rRNA sequencing, Bacteria

## Abstract

**Background:**

Use of skin personal care products on a regular basis is nearly ubiquitous, but their effects on molecular and microbial diversity of the skin are unknown. We evaluated the impact of four beauty products (a facial lotion, a moisturizer, a foot powder, and a deodorant) on 11 volunteers over 9 weeks.

**Results:**

Mass spectrometry and 16S rRNA inventories of the skin revealed decreases in chemical as well as in bacterial and archaeal diversity on halting deodorant use. Specific compounds from beauty products used before the study remain detectable with half-lives of 0.5–1.9 weeks. The deodorant and foot powder increased molecular, bacterial, and archaeal diversity, while arm and face lotions had little effect on bacterial and archaeal but increased chemical diversity. Personal care product effects last for weeks and produce highly individualized responses, including alterations in steroid and pheromone levels and in bacterial and archaeal ecosystem structure and dynamics.

**Conclusions:**

These findings may lead to next-generation precision beauty products and therapies for skin disorders.

**Electronic supplementary material:**

The online version of this article (10.1186/s12915-019-0660-6) contains supplementary material, which is available to authorized users.

## Background

The human skin is the most exposed organ to the external environment and represents the first line of defense against external chemical and microbial threats. It harbors a microbial habitat that is person-specific and varies considerably across the body surface [1–4]. Recent findings suggested an association between the use of antiperspirants or make-up and skin microbiota composition [5–7]. However, these studies were performed for a short period (7–10 days) and/or without washing out the volunteers original personal care products, leading to incomplete evaluation of microbial alterations because the process of skin turnover takes 21–28 days [5–9]. It is well-established that without intervention, most adult human microbiomes, skin or other microbiomes, remain stable compared to the differences between individuals [3, 10–16].

Although the skin microbiome is stable for years [10], little is known about the molecules that reside on the skin surface or how skin care products influence this chemistry [17, 18]. Mass spectrometry can be used to detect host molecules, personalized lifestyles including diet, medications, and personal care products [18, 19]. However, although the impact of short-term dietary interventions on the gut microbiome has been assessed [20, 21], no study has yet tested how susceptible the skin chemistry and Microbiome are to alterations in the subjects’ personal care product routine.

In our recent metabolomic/microbiome 3D cartography study [18], we observed altered microbial communities where specific skin care products were present. Therefore, we hypothesized that these products might shape specific skin microbial communities by changing their chemical environment. Some beauty product ingredients likely promote or inhibit the growth of specific bacteria: for example, lipid components of moisturizers could provide nutrients and promote the growth of lipophilic bacteria such as *Staphylococcus* and *Propionibacterium* [18, 22, 23]. Understanding both temporal variations of the skin microbiome and chemistry is crucial for testing whether alterations in personal habits can influence the human skin ecosystem and, perhaps, host health. To evaluate these variations, we used a multi-omics approach integrating metabolomics and microbiome data from skin samples of 11 healthy human individuals. Here, we show that many compounds from beauty products persist on the skin for weeks following their use, suggesting a long-term contribution to the chemical environment where skin microbes live. Metabolomics analysis reveals temporal trends correlated to discontinuing and resuming the use of beauty products and characteristic of variations in molecular composition of the skin. Although highly personalized, as seen with the microbiome, the chemistry, including hormones and pheromones such as androstenone and androsterone, were dramatically altered. Similarly, by experimentally manipulating the personal care regime of participants, bacterial and molecular diversity and structure are altered, particularly for the armpits and feet. Interestingly, a high person-to-person molecular and bacterial variability is maintained over time even though personal care regimes were modified in exactly the same way for all participants.

## Results

### Skin care and hygiene products persist on the skin

**Fig. 1:**
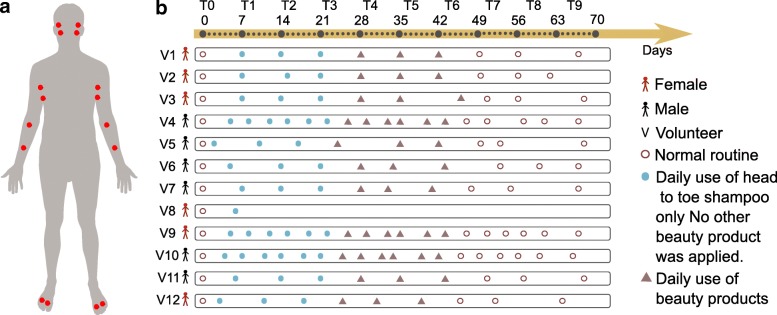
Study design and representation of changes in personal care regime over the course of 9 weeks. **a** Six males and six females were recruited and sampled using swabs on two locations from each body part (face, armpits, front forearms, and between toes) on the right and left side. The locations sampled were the face—upper cheek bone and lower jaw, armpit—upper and lower area, arm—front of elbow (antecubitis) and forearm (antebrachium), and feet—in between the first and second toe and third and fourth toe. Volunteers were asked to follow specific instructions for the use of skin care products. **b** Following the use of their personal skin care products (brown circles), all volunteers used only the same head to toe shampoo during the first 3 weeks (week 1–week 3) and no other beauty product was applied (solid blue circle). The following 3 weeks (week 4–week 6), four selected commercial beauty products were applied daily by all volunteers on the specific body part (deodorant antiperspirant for the armpits, soothing foot powder for the feet between toes, sunscreen for the face, and moisturizer for the front forearm) (triangles) and continued to use the same shampoo. During the last 3 weeks (week 7–week 9), all volunteers went back to their normal routine and used their personal beauty products (circles). Samples were collected once a week (from day 0 to day 68—10 timepoints from T0 to T9) for volunteers 1, 2, 3, 4, 5, 6, 7, 9, 10, 11, and 12, and on day 0 and day 6 for volunteer 8, who withdraw from the study after day 6. For 3 individuals (volunteers 4, 9, 10), samples were collected twice a week (19 timepoints total). Samples collected for 11 volunteers during 10 timepoints: 11 volunteers × 10 timepoints × 4 samples × 4 body sites = 1760. Samples collected from 3 selected volunteers during 9 additional timepoints: 3 volunteers × 9 timepoints × 4 samples × 4 body sites = 432. See also the “Subject recruitment and sample collection” section in the “Methods” section

**Fig. 2:**
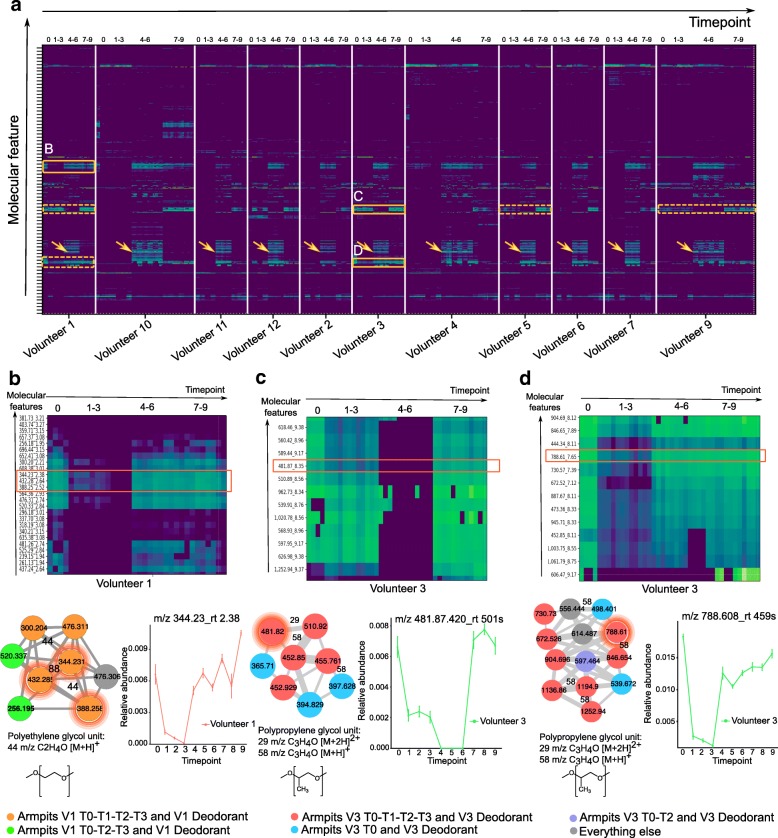
Monitoring the persistence of personal care product ingredients in the armpits over a 9-week period. **a** Heatmap representation of the most abundant molecular features detected in the armpits of all individuals during the four phases (0: initial, 1–3: no beauty products, 4–6: common products, and 7–9: personal products). Green color in the heatmap represents the highest molecular abundance and blue color the lowest one. Orange boxes with plain lines represent enlargement of cluster of molecules that persist on the armpits of volunteer 1 (**b**) and volunteer 3 (**c**, **d**). Orange clusters with dotted lines represent same clusters of molecules found on the armpits of other volunteers. Orange arrows represent the cluster of compounds characteristic of the antiperspirant used during T4–T6. **b** Polyethylene glycol (PEG) molecular clusters that persist on the armpits of individual 1. The molecular subnetwork, representing molecular families [24], is part of a molecular network (http://gnps.ucsd.edu/ProteoSAFe/status.jsp?task=f5325c3b278a46b29e8860ec5791d5ad) generated from MS/MS data collected from the armpits of volunteer 1 (T0–T3) MSV000081582 and MS/MS data collected from the deodorant used by volunteer 1 before the study started (T0) MSV000081580. **c**, **d** Polypropylene glycol (PPG) molecular families that persist on the armpits of individual 3, along with the corresponding molecular subnetwork that is part of the molecular network accessible here http://gnps.ucsd.edu/ProteoSAFe/status.jsp?task=aaa1af68099d4c1a87e9a09f398fe253. Subnetworks were generated from MS/MS data collected from the armpits of volunteer 3 (T0–T3) MSV000081582 and MS/MS data collected from the deodorant used by volunteer 3 at T0 MSV000081580. The network nodes were annotated with colors. Nodes represent MS/MS spectra found in armpit samples of individual 1 collected during T0, T1, T2, and T3 and in personal deodorant used by individual 1 (orange nodes); armpit samples of individual 1 collected during T0, T2, and T3 and personal deodorant used by individual 1 (green nodes); armpit samples of individual 3 collected during T0, T1, T2, and T3 and in personal deodorant used by individual 3 (red nodes); armpit samples of individual 3 collected during T0 and in personal deodorant used by individual 3 (blue nodes); and armpit samples of individual 3 collected during T0 and T2 and in personal deodorant used by individual 3 (purple nodes). Gray nodes represent everything else. Error bars represent standard error of the mean calculated at each timepoint from four armpit samples collected from the right and left side of each individual separately. See also Additional file 1: Figure S1

Systematic strategies to influence both the skin chemistry and microbiome have not yet been investigated. The outermost layer of the skin turns over every 3 to 4 weeks [8, 9]. How the microbiome and chemistry are influenced by altering personal care and how long the chemicals of personal care products persist on the skin are essentially uncharacterized. In this study, we collected samples from skin of 12 healthy individuals—six males and six females—over 9 weeks. One female volunteer had withdrawn due to skin irritations that developed, and therefore, we describe the remaining 11 volunteers. Samples were collected from each arm, armpit, foot, and face, including both the right and left sides of the body (Fig. 1a). All participants were asked to adhere to the same daily personal care routine during the first 6 weeks of this study (Fig. 1b). The volunteers were asked to refrain from using any personal care product for weeks 1–3 except a mild body wash (Fig. 1b). During weeks 4–6, in addition to the body wash, participants were asked to apply selected commercial skin care products at specific body parts: a moisturizer on the arm, a sunscreen on the face, an antiperspirant on the armpits, and a soothing powder on the foot (Fig. 1b). To monitor adherence of participants to the study protocol, molecular features found in the antiperspirant, facial lotion, moisturizer, and foot powder were directly tracked with mass spectrometry from the skin samples. For all participants, the mass spectrometry data revealed the accumulation of specific beauty product ingredients during weeks 4–6 (Additional file 1: Figure S1A-I, Fig. 2a orange arrows). Examples of compounds that were highly abundant during T4–T6 in skin samples are avobenzone (Additional file 1: Figure S1A), dexpanthenol (Additional file 1: Figure S1B), and benzalkonium chloride (Additional file 1: Figure S1C) from the facial sunscreen; trehalose 6-phosphate (Additional file 1: Figure S1D) and glycerol stearate (Additional file 1: Figure S1E) from the moisturizer applied on arms; indolin (Additional file 1: Figure S1F) and an unannotated compound (*m/z* 233.9, rt 183.29 s) (Additional file 1: Figure S1G) from the foot powder; and decapropylene glycol (Additional file 1: Figure S1H) and nonapropylene glycol (Additional file 1: Figure S1I) from the antiperspirant. These results suggest that there is likely a compliance of all individuals to study requirements and even if all participants confirmed using each product every day, the amount of product applied by each individual may vary. Finally, for weeks 7–9, the participants were asked to return to their normal routine by using the same personal care products they used prior to the study. In total, excluding all blanks and personal care products themselves, we analyzed 2192 skin samples for both metabolomics and microbiome analyses.

To understand how long beauty products persist on the skin, we monitored compounds found in deodorants used by two volunteers—female 1 and female 3—before the study (T0), over the first 3 weeks (T1–T3) (Fig. 1b). During this phase, all participants used exclusively the same body wash during showering, making it easier to track ingredients of their personal care products. The data in the first 3 weeks (T1–T3) revealed that many ingredients of deodorants used on armpits (Fig. 2a) persist on the skin during this time and were still detected during the first 3 weeks or at least during the first week following the last day of use. Each of the compounds detected in the armpits of individuals exhibited its own unique half-life. For example, the polyethylene glycol (PEG)-derived compounds *m/z* 344.227, rt 143 s (Fig. 2b, S1J); *m/z* 432.279, rt 158 s (Fig. 2b, S1K); and *m/z* 388.253, rt 151 s (Fig. 2b, S1L) detected on armpits of volunteer 1 have a calculated half-life of 0.5 weeks (Additional file 1: Figure S1J-L, all *p* values < 1.81e−07), while polypropylene glycol (PPG)-derived molecules *m/z* 481.87, rt 501 s (Fig. 2c, S1M); *m/z* 560.420, rt 538 s (Fig. 2c, S1N); *m/z* 788.608, rt 459 s (Fig. 2d, S1O); *m/z* 846.650, rt 473 s (Fig. 2d, S1P); and *m/z* 444.338, rt 486 s (Fig. 2d, S1Q) found on armpits of volunteers 3 and 1 (Fig. 2a) have a calculated half-life ranging from 0.7 to 1.9 weeks (Additional file 1: Figure S1M-Q, all *p* values < 0.02), even though they originate from the same deodorant used by each individual. For some ingredients of deodorant used by volunteer 3 on time 0 (Additional file 1: Figure S1M, N), a decline was observed during the first week, then little to no traces of these ingredients were detected during weeks 4–6 (T4–T6), then finally these ingredients reappear again during the last 3 weeks of personal product use (T7–T9). This suggests that these ingredients are present exclusively in the personal deodorant used by volunteer 3 before the study. Because a similar deodorant (Additional file 1: Figure S1O-Q) and a face lotion (Additional file 1: Figure S1R) was used by volunteer 3 and volunteer 2, respectively, prior to the study, there was no decline or absence of their ingredients during weeks 4–6 (T4–T6).

Polyethylene glycol compounds (Additional file 1: Figure S1J-L) wash out faster from the skin than polypropylene glycol (Additional file 1: Figure S1M-Q)(HL ~ 0.5 weeks vs ~ 1.9 weeks) and faster than fatty acids used in lotions (HL ~ 1.2 weeks) (Additional file 1: Figure S1R), consistent with their hydrophilic (PEG) and hydrophobic properties (PPG and fatty acids) [25, 26]. This difference in hydrophobicity is also reflected in the retention time as detected by mass spectrometry. Following the linear decrease of two PPG compounds from T0 to T1, they accumulated noticeably during weeks 2 and 3 (Additional file 1: Figure S1M, N). This accumulation might be due to other sources of PPG such as the body wash used during this period or the clothes worn by person 3. Although PPG compounds were not listed in the ingredient list of the shampoo, we manually inspected the LC-MS data collected from this product and confirmed the absence of PPG compounds in the shampoo. The data suggest that this trend is characteristic of accumulation of PPG from additional sources. These could be clothes, beds, or sheets, in agreement with the observation of these molecules found in human habitats [27] but also in the public GNPS mass spectrometry dataset MSV000079274 that investigated the chemicals from dust collected from 1053 mattresses of children.

### Temporal molecular and bacterial diversity in response to personal care use

**Fig. 3:**
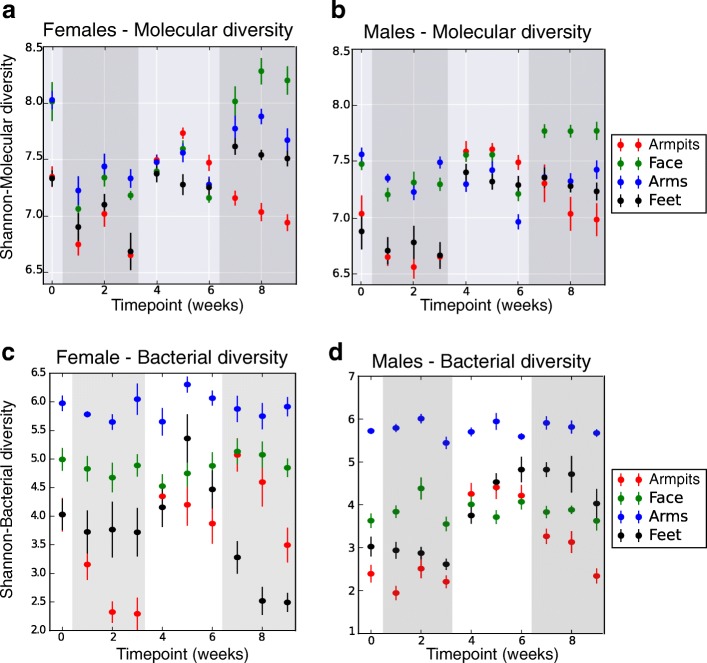
Molecular and bacterial diversity over a 9-week period, comparing samples based on their molecular (UPLC-Q-TOF-MS) or bacterial (16S rRNA amplicon) profiles. Molecular and bacterial diversity using the Shannon index was calculated from samples collected from each body part at each timepoint, separately for female (*n* = 5) and male (*n* = 6) individuals. Error bars represent standard error of the mean calculated at each timepoint, from up to four samples collected from the right and left side of each body part, of females (*n* = 5) and males (*n* = 6) separately. **a**, **b** Molecular alpha diversity measured using the Shannon index from five females (left panel) and six males (right panel), over 9 weeks, from four distinct body parts (armpits, face, arms, feet). **c**, **d** Bacterial alpha diversity measured using the Shannon index, from skin samples collected from five female (left panel) and six male individuals (right panel), over 9 weeks, from four distinct body parts (armpits, face, arms, feet). See also Additional file 1: Figure S2

To assess the effect of discontinuing and resuming the use of skin care products on molecular and microbiota dynamics, we first evaluated their temporal diversity. Skin sites varied markedly in their initial level (T0) of molecular and bacterial diversity, with higher molecular diversity at all sites for female participants compared to males (Fig. 3a, b, Wilcoxon rank-sum-WR test, *p* values ranging from 0.01 to 0.0001, from foot to arm) and higher bacterial diversity in face (WR test, *p* = 0.0009) and armpits (WR test, *p* = 0.002) for females (Fig. 3c, d). Temporal diversity was similar across the right and left sides of each body site of all individuals (WR test, molecular diversity: all *p* values > 0.05; bacterial diversity: all *p* values > 0.20). The data show that refraining from using beauty products (T1–T3) leads to a significant decrease in molecular diversity at all sites (Fig. 3a, b, WR test, face: *p* = 8.29e−07, arm: *p* = 7.08e−09, armpit: *p* = 1.13e−05, foot: *p* = 0.002) and bacterial diversity mainly in armpits (WR test, *p* = 0.03) and feet (WR test, *p* = 0.04) (Fig. 3c, d). While molecular diversity declined (Fig. 3a, b) for arms and face, bacterial diversity (Fig. 3c, d) was less affected in the face and arms when participants did not use skin care products (T1–T3). The molecular diversity remained stable in the arms and face of female participants during common beauty products use (T4–T6) to immediately increase as soon as the volunteers went back to their normal routines (T7–T9) (WR test, *p* = 0.006 for the arms and face)(Fig. 3a, b). A higher molecular (Additional file 1: Figure S2A) and community (Additional file 1: Figure S2B) diversity was observed for armpits and feet of all individuals during the use of antiperspirant and foot powder (T4–T6) (WR test, molecular diversity: armpit *p* = 8.9e−33, foot *p* = 1.03e−11; bacterial diversity: armpit *p* = 2.14e−28, foot *p* = 1.26e−11), followed by a molecular and bacterial diversity decrease in the armpits when their regular personal beauty product use was resumed (T7–T9) (bacterial diversity: WR test, *p* = 4.780e−21, molecular diversity: WR test, *p* = 2.159e−21). Overall, our data show that refraining from using beauty products leads to lower molecular and bacterial diversity, while resuming the use increases their diversity. Distinct variations between male and female molecular and community richness were perceived at distinct body parts (Fig. 3a–d). Although the chemical diversity of personal beauty products does not explain these variations (Additional file 1: Figure S2C), differences observed between males and females may be attributed to many environmental and lifestyle factors including different original skin care and different frequency of use of beauty products (Additional file 2: Table S1), washing routines, and diet.

### Longitudinal variation of skin metabolomics signatures

**Fig. 4:**
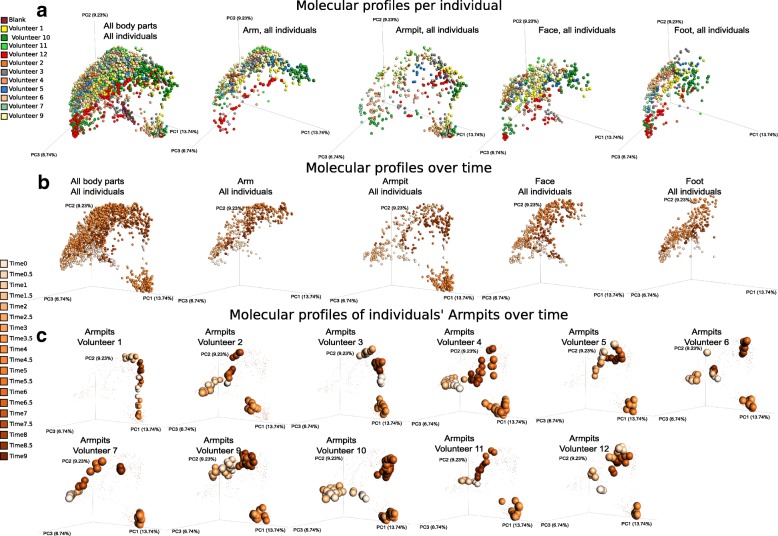
Individualized influence of beauty product application on skin metabolomics profiles over time. **a** Multivariate statistical analysis (principal coordinate analysis (PCoA)) comparing mass spectrometry data collected over 9 weeks from the skin of 11 individuals, all body parts, combined (first plot from the left) and then displayed separately (arm, armpits, face, feet). Color scale represents volunteer ID. The PCoA was calculated on all samples together, and subsets of the data are shown in this shared space and the other panels. **b** The molecular profiles collected over 9 weeks from all body parts, combined then separately (arm, armpits, face, feet). **c** Representative molecular profiles collected over 9 weeks from armpits of 11 individuals (volunteers 1, 2, 3, 4, 5, 6, 7, 9, 10, 11, 12). Color gradient in **b** and **c** represents timepoints (time 0 to time 9), ranging from the lightest orange color to the darkest one that represent the earliest (time 0) to the latest (time 9) timepoint, respectively. 0.5 timepoints represent additional timepoints where three selected volunteers were samples (volunteers 4, 9, and 10). PCoA plots were generated using the Bray–Curtis dissimilarity matrix and visualized in Emperor [28]. See also Additional file 1: Figure S3

To gain insights into temporal metabolomics variation associated with beauty product use, chemical inventories collected over 9 weeks were subjected to multivariate analysis using the widely used Bray–Curtis dissimilarity metric (Fig. 4a–c, S3A). Throughout the 9-week period, distinct molecular signatures were associated to each specific body site: arm, armpit, face, and foot (Additional file 1: Figure S3A, Adonis test, *p* < 0.001, *R*^2^ 0.12391). Mass spectrometric signatures displayed distinct individual trends at each specific body site (arm, armpit, face, and foot) over time, supported by their distinct locations in PCoA (principal coordinate analysis) space (Fig. 4a, b) and based on the Bray–Curtis distances between molecular profiles (Additional file 1: Figure S3B, WR test, all *p* values < 0.0001 from T0 through T9). This suggests a high molecular inter-individual variability over time despite similar changes in personal care routines. Significant differences in molecular patterns associated to ceasing (T1–T3) (Fig. 4b, Additional file 1: Figure S3C, WR test, T0 vs T1–T3 *p* < 0.001) and resuming the use of common beauty products (T4–T6) (Additional file 1: Figure S3C) were observed in the arm, face, and foot (Fig. 4b), although the armpit exhibited the most pronounced changes (Fig. 4b, Additional file 1: Figure S3D, E, random forest highlighting that 100% of samples from each phase were correctly predicted). Therefore, we focused our analysis on this region. Molecular changes were noticeable starting the first week (T1) of discontinuing beauty product use. As shown for armpits in Fig. 4c, these changes at the chemical level are specific to each individual, possibly due to the extremely personalized lifestyles before the study and match their original use of deodorant. Based on the initial use of underarm products (T0) (Additional file 2: Table S1), two groups of participants can be distinguished: a group of five volunteers who used stick deodorant as evidenced by the mass spectrometry data and another group of volunteers where we found few or no traces suggesting they never or infrequently used stick deodorants (Additional file 2: Table S1). Based on this criterion, the chemical trends shown in Fig. 4c highlight that individuals who used stick deodorant before the beginning of the study (volunteers 1, 2, 3, 9, and 12) displayed a more pronounced shift in their armpits’ chemistries as soon as they stopped using deodorant (T1–T3), compared to individuals who had low detectable levels of stick deodorant use (volunteers 4, 6, 7, and 10), or “rarely-to-never” (volunteers 5 and 11) use stick deodorants as confirmed by the volunteers (Additional file 1: Figure S3F, WR test, T0 vs T1–T3 all *p* values < 0.0001, with greater distance for the group of volunteers 1, 2, 3, 9, and 12, compared to volunteers 4, 5, 6, 7, 10, and 11). The most drastic shift in chemical profiles was observed during the transition period, when all participants applied the common antiperspirant on a daily basis (T4–T6) (Additional file 1: Figure S3D, E). Finally, the molecular profiles became gradually more similar to those collected before the experiment (T0) as soon as the participants resumed using their personal beauty products (T7–T9) (Additional file 1: Figure S3C), although traces of skin care products did last through the entire T7–T9 period in people who do not routinely apply these products (Fig. 4c).

Comparing chemistries detected in armpits at the end timepoints—when no products were used (T3) and during product use (T6)—revealed distinct molecular signatures characteristic of each phase (random forest highlighting that 100% of samples from each group were correctly predicted, see Additional file 1: Figure S3D, E). Because volunteers used the same antiperspirant during T4–T6, molecular profiles converged during that time despite individual patterns at T3 (Fig. 4b, c, Additional file 1: Figure S3D). These distinct chemical patterns reflect the significant impact of beauty products on skin molecular composition. Although these differences may in part be driven by beauty product ingredients detected on the skin (Additional file 1: Figure S1), we anticipated that additional host- and microbe-derived molecules may also be involved in these molecular changes.

To characterize the chemistries that vary over time, we used molecular networking, a MS visualization approach that evaluates the relationship between MS/MS spectra and compares them to reference MS/MS spectral libraries of known compounds [29, 30]. We recently showed that molecular networking can successfully organize large-scale mass spectrometry data collected from the human skin surface [18, 19]. Briefly, molecular networking uses the MScluster algorithm [31] to merge all identical spectra and then compares and aligns all unique pairs of MS/MS spectra based on their similarities where 1.0 indicates a perfect match. Similarities between MS/MS spectra are calculated using a similarity score, and are interpreted as molecular families [19, 24, 32–34]. Here, we used this method to compare and characterize chemistries found in armpits, arms, face, and foot of 11 participants. Based on MS/MS spectral similarities, chemistries highlighted through molecular networking (Additional file 1: Figure S4A) were associated with each body region with 8% of spectra found exclusively in the arms, 12% in the face, 14% in the armpits, and 2% in the foot, while 18% of the nodes were shared between all four body parts and the rest of spectra were shared between two body sites or more (Additional file 1: Figure S4B). Greater spectral similarities were highlighted between armpits, face, and arm (12%) followed by the arm and face (9%) (Additional file 1: Figure S4B).

**Fig. 5:**
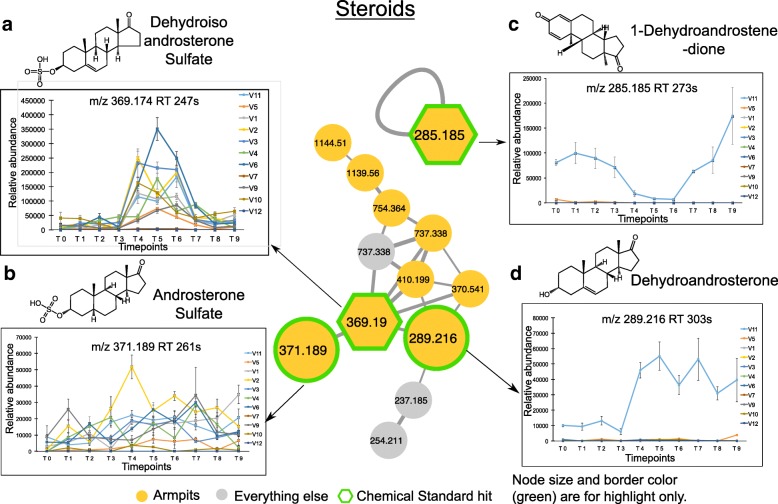
Underarm steroids and their longitudinal abundance. **a**–**d** Steroid molecular families in the armpits and their relative abundance over a 9-week period. Molecular networking was applied to characterize chemistries from the skin of 11 healthy individuals. The full network is shown in Additional file 1: Figure S4A, and networking parameters can be found here http://gnps.ucsd.edu/ProteoSAFe/status.jsp?task=284fc383e4c44c4db48912f01905f9c5 for MS/MS datasets MSV000081582. Each node represents a consensus of a minimum of 3 identical MS/MS spectra. Yellow nodes represent MS/MS spectra detected in armpits samples. Hexagonal shape represents MS/MS spectra match between skin samples and chemical standards. Plots are representative of the relative abundance of each compound over time, calculated separately from LC-MS1 data collected from the armpits of each individual. Steroids detected in armpits are **a**, dehydroisoandrosterone sulfate (*m/z* 369.190, rt 247 s), **b** androsterone sulfate (*m/z* 371.189, rt 261 s), **c** 1-dehydroandrostenedione (*m/z* 285.185, rt 273 s), and **d** dehydroandrosterone (*m/z* 289.216, rt 303 s). Relative abundance over time of each steroid compound is represented. Error bars represent the standard error of the mean calculated at each timepoint from four armpit samples from the right and left side of each individual separately. See also Additional file 1: Figures S4-S8

Molecules were annotated with Global Natural Products Social Molecular Networking (GNPS) libraries [29], using accurate parent mass and MS/MS fragmentation patterns, according to level 2 or 3 of annotation defined by the 2007 metabolomics standards initiative [35]. Through annotations, molecular networking revealed that many compounds derived from steroids (Fig. 5a–d), bile acids (Additional file 1: Figure S5A-D), and acylcarnitines (Additional file 1: Figure S5E-F) were exclusively detected in the armpits. Using authentic standards, the identity of some pheromones and bile acids were validated to a level 1 identification with matched retention times (Additional file 1: Figure S6B, S7A, C, D). Other steroids and bile acids were either annotated using standards with identical MS/MS spectra but slightly different retention times (Additional file 1: Figure S6A) or annotated with MS/MS spectra match with reference MS/MS library spectra (Additional file 1: Figure S6C, D, S7B, S6E-G). These compounds were therefore classified as level 3 [35]. Acylcarnitines were annotated to a family of possible acylcarnitines (we therefore classify as level 3), as the positions of double bonds or *cis* vs *trans* configurations are unknown (Additional file 1: Figure S8A, B).

Among the steroid compounds, several molecular families were characterized: androsterone (Fig. 5a, b, d), androstadienedione (Fig. 5c), androstanedione (Additional file 1: Figure S6E), androstanolone (Additional file 1: Figure S6F), and androstenedione (Additional file 1: Figure S6G). While some steroids were detected in the armpits of several individuals, such as dehydroisoandrosterone sulfate (*m/z* 369.19, rt 247 s) (9 individuals) (Fig. 5a, Additional file 1: Figure S6A), androsterone sulfate (*m/z* 371.189, rt 261 s) (9 individuals) (Fig. 5b, Additional file 1: Figure S6C), and 5-alpha-androstane-3,17-dione (*m/z* 271.205, rt 249 s) (9 individuals) (Additional file 1: Figure S6E), other steroids including 1-dehydroandrostenedione (*m/z* 285.185, rt 273 s) (Fig. 5c, Additional file 1: Figure S6B), dehydroandrosterone (*m/z* 289.216, rt 303 s) (Fig. 5d, Additional file 1: Figure S6D), and 5-alpha-androstan-17.beta-ol-3-one (*m/z* 291.231, rt 318 s) (Additional file 1: Figure S6F) were only found in the armpits of volunteer 11 and 4-androstene-3,17-dione (*m/z* 287.200, rt 293 s) in the armpits of volunteer 11 and volunteer 5, both are male that never applied stick deodorants (Additional file 1: Figure S6G). Each molecular species exhibited a unique pattern over the 9-week period. The abundance of dehydroisoandrosterone sulfate (Fig. 5a, WR test, *p* < 0.01 for 7 individuals) and dehydroandrosterone (Fig. 5a, WR test, *p* = 0.00025) significantly increased during the use of antiperspirant (T4–T6), while androsterone sulfate (Fig. 5b) and 5-alpha-androstane-3,17-dione (Additional file 1: Figure S6E) display little variation over time. Unlike dehydroisoandrosterone sulfate (Fig. 5a) and dehydroandrosterone (Fig. 5d), steroids including 1-dehydroandrostenedione (Fig. 5c, WR test, *p* = 0.00024) and 4-androstene-3,17-dione (Additional file 1: Figure S6G, WR test, *p* = 0.00012) decreased in abundance during the 3 weeks of antiperspirant application (T4–T6) in armpits of male 11, and their abundance increased again when resuming the use of his normal skin care routines (T7–T9). Interestingly, even within the same individual 11, steroids were differently impacted by antiperspirant use as seen for 1-dehydroandrostenedione that decreased in abundance during T4–T6 (Fig. 5c, WR test, *p* = 0.00024), while dehydroandrosterone increased in abundance (Fig. 5d, WR test, *p* = 0.00025), and this increase was maintained during the last 3 weeks of the study (T7–T9).

In addition to steroids, many bile acids (Additional file 1: Figure S5A-D) and acylcarnitines (Additional file 1: Figure S5E-F) were detected on the skin of several individuals through the 9-week period. Unlike taurocholic acid found only on the face (Additional file 1: Figures S5A, S7A) and tauroursodeoxycholic acid detected in both armpits and arm samples (Additional file 1: Figures S5B, S7B), other primary bile acids such as glycocholic (Additional file 1: Figures S5C, S7C) and chenodeoxyglycocholic acid (Additional file 1: Figures S5D, S7D) were exclusively detected in the armpits. Similarly, acylcarnitines were also found either exclusively in the armpits (hexadecanoyl carnitines) (Additional file 1: Figures S5E, S8A) or in the armpits and face (tetradecenoyl carnitine) (Additional file 1: Figures S5F, S8B) and, just like the bile acids, they were also stably detected during the whole 9-week period.

### Bacterial communities and their variation over time

**Fig. 6:**
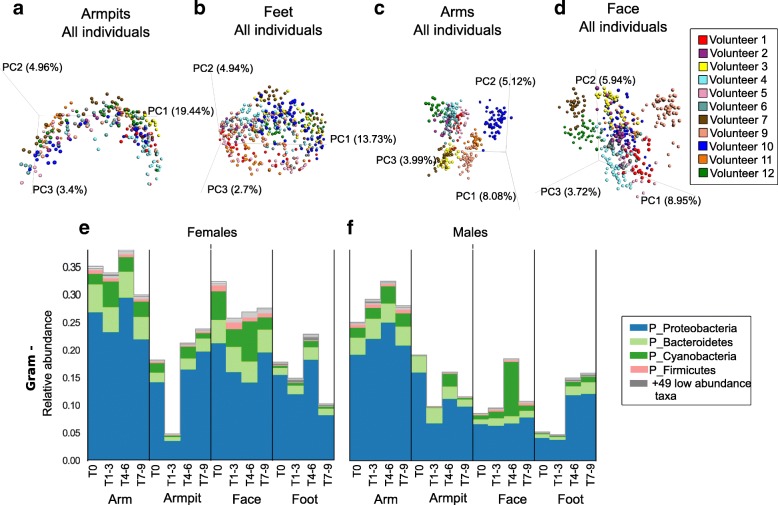
Longitudinal variation of skin bacterial communities in association with beauty product use. **a**-**d** Bacterial profiles collected from skin samples of 11 individuals, over 9 weeks, from four distinct body parts a) armpits, b) feet, c) arms and d) face, using multivariate statistical analysis (Principal Coordinates Analysis PCoA) and unweighted Unifrac metric. Each color represents bacterial samples collected from an individual. PCoA were calculated separately for each body part. **e**, **f** Representative Gram-negative (Gram -) bacteria collected from arms, armpits, face and feet of e) female and f) male participants. See also Additional file 1: Figure S9A, B showing Gram-negative bacterial communities represented at the genus level

Having demonstrated the impact of beauty products on the chemical makeup of the skin, we next tested the extent to which skin microbes are affected by personal care products. We assessed temporal variation of bacterial communities detected on the skin of healthy individuals by evaluating dissimilarities of bacterial collections over time using unweighted UniFrac distance [36] and community variation at each body site in association to beauty product use [3, 15, 37]. Unweighted metrics are used for beta diversity calculations because we are primarily concerned with changes in community membership rather than relative abundance. The reason for this is that skin microbiomes can fluctuate dramatically in relative abundance on shorter timescales than that assessed here. Longitudinal variations were revealed for the armpits (Fig. 6a) and feet microbiome by their overall trend in the PCoA plots (Fig. 6b), while the arm (Fig. 6c) and face (Fig. 6d) displayed relatively stable bacterial profiles over time. As shown in Fig. 6a–d, although the microbiome was site-specific, it varied more between individuals and this inter-individual variability was maintained over time despite same changes in personal care routine (WR test, all *p* values at all timepoints < 0.05, T5 *p* = 0.07), in agreement with previous findings that individual differences in the microbiome are large and stable over time [3, 4, 10, 37]. However, we show that shifts in the microbiome can be induced by changing hygiene routine and therefore skin chemistry. Changes associated with using beauty products (T4–T6) were more pronounced for the armpits (Fig. 6a, WR test, *p* = 1.61e−52) and feet (Fig. 6b, WR test, *p* = 6.15e−09), while little variations were observed for the face (Fig. 6d, WR test, *p* = 1.402.e−83) and none for the arms (Fig. 6c, WR test, *p* = 0.296).

A significant increase in abundance of Gram-negative bacteria including the phyla *Proteobacteria* and *Bacteroidetes* was noticeable for the armpits and feet of both females (Fig. 6e; Mann–Whitney *U*, *p* = 8.458e−07) and males (Fig. 6f; Mann–Whitney *U*, *p* = 0.0004) during the use of antiperspirant (T4–T6), while their abundance remained stable for the arms and face during that time (Fig. 6e, f; female arm *p* = 0.231; female face *p* value = 0.475; male arm *p*= 0.523;male face *p* = 6.848751e−07). These Gram-negative bacteria include *Acinetobacter* and *Paracoccus* genera that increased in abundance in both armpits and feet of females (Additional file 1: Figure S9A), while a decrease in abundance of *Enhydrobacter* was observed in the armpits of males (Additional file 1: Figure S9B). Cyanobacteria, potentially originating from plant material (Additional file 1: Figure S9C) also increased during beauty product use (T4–T6) especially in males, in the armpits and face of females (Fig. 6e) and males (Fig. 6f). Interestingly, although chloroplast sequences (which group phylogenetically within the cyanobacteria [38]) were only found in the facial cream (Additional file 1: Figure S9D), they were detected in other locations as well (Fig. 6e, f. S9E, F), highlighting that the application of a product in one region will likely affect other regions of the body. For example, when showering, a face lotion will drip down along the body and may be detected on the feet. Indeed, not only did the plant material from the cream reveal this but also the shampoo used for the study for which molecular signatures were readily detected on the feet as well (Additional file 1: Figure S10A). Minimal average changes were observed for Gram-positive organisms (Additional file 1: Figure S10B, C), although in some individuals the variation was greater than others (Additional file 1: Figure S10D, E) as discussed for specific Gram-positive taxa below.

**Fig. 7:**
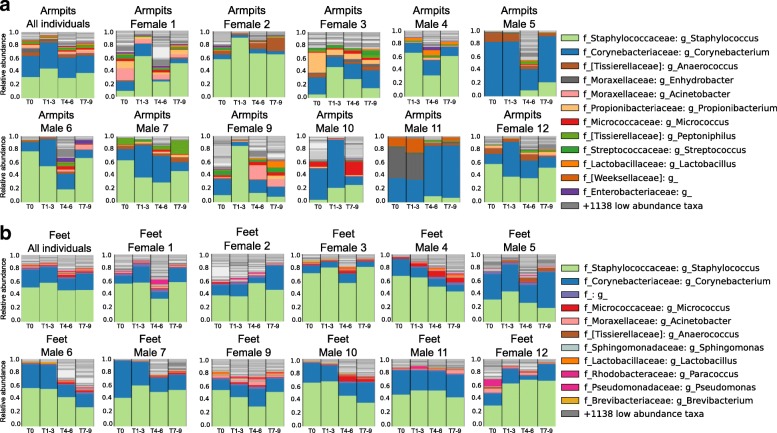
Person-to-person bacterial variabilities over time in the armpits and feet. **a** Armpit microbiome changes when stopping personal care product use, then resuming. Armpit bacterial composition of the 11 volunteers combined, then separately, (female 1, female 2, female 3, male 4, male 5, male 6, male 7, female 9, male 10, male 11, female 12) according to the four periods within the experiment. **b** Feet bacterial variation over time of the 12 volunteers combined, then separately (female 1, female 2, female 3, male 4, male 5, male 6, male 7, female 9, male 10, male 11, female 12) according to the four periods within the experiment. See also Additional file 1: Figure S9-S13

At T0, the armpit’s microflora was dominated by *Staphylococcus* (26.24%, 25.11% of sequencing reads for females and 27.36% for males) and *Corynebacterium* genera (26.06%, 17.89% for females and 34.22% for males) (Fig. 7a—first plot from left and Additional file 1: Figure S10D, E). They are generally known as the dominant armpit microbiota and make up to 80% of the armpit microbiome [39, 40]. When no deodorants were used (T1–T3), an overall increase in relative abundance of *Staphylococcus* (37.71%, 46.78% for females and 30.47% for males) and *Corynebacterium* (31.88%, 16.50% for females and 44.15% for males) genera was noticeable (WR test, *p* < 3.071e−05) (Fig. 7a—first plot from left), while the genera *Anaerococcus* and *Peptoniphilus* decreased in relative abundance (WR test, *p* < 0.03644) (Fig. 7a—first plot from left and Additional file 1: Figure S10D, E). When volunteers started using antiperspirants (T4–T6), the relative abundance of *Staphylococcus* (37.71%, 46.78% females and 30.47% males, to 21.71%, 25.02% females and 19.25% males) and *Corynebacterium* (31.88%, 16.50% females and 44.15% males, to 15.83%, 10.76% females and 19.60% males) decreased (WR test, *p* < 3.071e−05) (Fig. 7a, Additional file 1: Figure S10D, E) and at the same time, the overall alpha diversity increased significantly (WR test, *p* = 3.47e−11) (Fig. 3c, d). The microbiota *Anaerococcus* (WR test, *p* = 0.0006018)*, Peptoniphilus* (WR test, *p* = 0.008639), and *Micrococcus* (WR test, *p* = 0.0377) increased significantly in relative abundance, together with a lot of additional low-abundant species that lead to an increase in Shannon alpha diversity (Fig. 3c, d). When participants went back to normal personal care products (T7–T9), the underarm microbiome resembled the original underarm community of T0 (WR test, *p* = 0.7274) (Fig. 7a). Because armpit bacterial communities are person-specific (inter-individual variability: WR test, all *p* values at all timepoints < 0.05, besides T5 *p* n.s), variation in bacterial abundance upon antiperspirant use (T4–T6) differ between individuals and during the whole 9-week period (Fig. 7a—taxonomic plots per individual). For example, the underarm microbiome of male 5 exhibited a unique pattern, where *Corynebacterium* abundance decreased drastically during the use of antiperspirant (82.74 to 11.71%, WR test, *p* = 3.518e−05) while in the armpits of female 9 a huge decrease in *Staphylococcus* abundance was observed (Fig. 7a) (65.19 to 14.85%, WR test, *p* = 0.000113). Unlike other participants, during T0–T3, the armpits of individual 11 were uniquely characterized by the dominance of a sequence that matched most closely to the *Enhydrobacter* genera*.* The transition to antiperspirant use (T4–T6) induces the absence of *Enhydrobacter* (30.77 to 0.48%, WR test, *p* = 0.01528) along with an increase of *Corynebacterium* abundance (26.87 to 49.74%, WR test, *p* = 0.1123) (Fig. 7a—male 11).

In addition to the armpits, a decline in abundance of *Staphylococcus* and *Corynebacterium* was perceived during the use of the foot powder (46.93% and 17.36%, respectively) compared to when no beauty product was used (58.35% and 22.99%, respectively) (WR test, *p* = 9.653e−06 and *p* = 0.02032, respectively), while the abundance of low-abundant foot bacteria significantly increased such as *Micrococcus* (WR test, *p* = 1.552e−08), *Anaerococcus* (WR test, *p* = 3.522e−13), *Streptococcus* (WR test, *p* = 1.463e−06), *Brevibacterium* (WR test, *p* = 6.561e−05), Moraxellaceae (WR test, *p* = 0.0006719), and *Acinetobacter* (WR test, *p* = 0.001487), leading to a greater bacterial diversity compared to other phases of the study (Fig. 7b first plot from left, Additional file 1: Figure S10D, E, Fig. 3c, d).

We further evaluated the relationship between the two omics datasets by superimposing the principal coordinates calculated from metabolome and microbiome data (Procrustes analysis) (Additional file 1: Figure S11) [34, 41, 42]. Metabolomics data were more correlated with patterns observed in microbiome data in individual 3 (Additional file 1: Figure S11C, Mantel test, *r* = 0.23, *p* < 0.001), individual 5 (Additional file 1: Figure S11E, *r* = 0.42, *p* < 0.001), individual 9 (Additional file 1: Figure S11H, *r* = 0.24, *p* < 0.001), individual 10 (Additional file 1: Figure S11I, *r* = 0.38, *p* < 0.001), and individual 11 (Additional file 1: Figure S11J, *r* = 0.35, *p* < 0.001) when compared to other individuals 1, 2, 4, 6, 7, and 12 (Additional file 1: Figure S11A, B, D, F, G, K, respectively) (Mantel test, all *r* < 0.2, all *p* values < 0.002, for volunteer 2 *p* n.s). Furthermore, these correlations were individually affected by ceasing (T1–T3) or resuming the use of beauty products (T4–T6 and T7–T9) (Additional file 1: Figure S11A-K).

Overall, metabolomics–microbiome correlations were consistent over time for the arms, face, and feet although alterations were observed in the arms of volunteers 7 (Additional file 1: Figure S11G) and 10 (Additional file 1: Figure S11I) and the face of volunteer 7 (Additional file 1: Figure S11G) during product use (T4–T6). Molecular–bacterial correlations were mostly affected in the armpits during antiperspirant use (T4–T6), as seen for volunteers male 7 (Additional file 1: Figure S11G) and 11 (Additional file 1: Figure S11J) and females 2 (Additional file 1: Figure S11B), 9 (Additional file 1: Figure S11H), and 12 (Additional file 1: Figure S11K). This perturbation either persisted during the last 3 weeks (Additional file 1: Figure S11D, E, H, I, K) when individuals went back to their normal routine (T7–T9) or resembled the initial molecular–microbial correlation observed in T0 (Additional file 1: Figure S11C, G, J). These alterations in molecular–bacterial correlation are driven by metabolomics changes during antiperspirant use as revealed by metabolomics shifts on the PCoA space (Additional file 1: Figure S11), partially due to the deodorant’s chemicals (Additional file 1: Figure S1J, K) but also to changes observed in steroid levels in the armpits (Fig. 5A, C, D, Additional file 1: Figure S6G), suggesting metabolome-dependant changes of the skin microbiome. In agreement with previous findings that showed efficient biotransformation of steroids by *Corynebacterium* [43, 44], our correlation analysis associates specific steroids that were affected by antiperspirant use in the armpits of volunteer 11 (Fig. 5c, d, Additional file 1: Figure S6G) with microbes that may produce or process them: 1-dehydroandrostenedione, androstenedione, and dehydrosterone with *Corynebacterium* (*r* = − 0.674, *p* = 6e−05; *r* = 0.671, *p* = 7e−05; *r* = 0.834, *p* < 1e−05, respectively) (Additional file 1: Figure S12A, B, C, respectively) and *Enhydrobacter* (*r* = 0.683, *p* = 4e−05; *r* = 0.581, *p* = 0.00095; *r* = 0.755, *p* < 1e−05 respectively) (Additional file 1: Figure S12D, E, F, respectively).

## Discussion

Despite the widespread use of skin care and hygiene products, their impact on the molecular and microbial composition of the skin is poorly studied. We established a workflow that examines individuals to systematically study the impact of such lifestyle characteristics on the skin by taking a broad look at temporal molecular and bacterial inventories and linking them to personal skin care product use. Our study reveals that when the hygiene routine is modified, the skin metabolome and microbiome can be altered, but that this alteration depends on product use and location on the body. We also show that like gut microbiome responses to dietary changes [20, 21], the responses are individual-specific.

We recently reported that traces of our lifestyle molecules can be detected on the skin days and months after the original application [18, 19]. Here, we show that many of the molecules associated with our personal skin and hygiene products had a half-life of 0.5 to 1.9 weeks even though the volunteers regularly showered, swam, or spent time in the ocean. Thus, a single application of some of these products has the potential to alter the microbiome and skin chemistry for extensive periods of time. Our data suggests that although host genetics and diet may play a role, a significant part of the resilience of the microbiome that has been reported [10, 45] is due to the resilience of the skin chemistry associated with personal skin and hygiene routines, or perhaps even continuous re-exposure to chemicals from our personal care routines that are found on mattresses, furniture, and other personal objects [19, 27, 46] that are in constant contact. Consistent with this observation is that individuals in tribal regions and remote villages that are infrequently exposed to the types of products used in this study have very different skin microbial communities [47, 48] and that the individuals in this study who rarely apply personal care products had a different starting metabolome. We observed that both the microbiome and skin chemistry of these individuals were most significantly affected by these products. This effect by the use of products at T4–T6 on the volunteers that infrequently used them lasted to the end phase of the study even though they went back to infrequent use of personal care products. What was notable and opposite to what the authors originally hypothesized is that the use of the foot powder and antiperspirant increased the diversity of microbes and that some of this diversity continued in the T7–T9 phase when people went back to their normal skin and hygiene routines. It is likely that this is due to the alteration in the nutrient availability such as fatty acids and moisture requirements, or alteration of microbes that control the colonization via secreted small molecules, including antibiotics made by microbes commonly found on the skin [49, 50].

We detected specific molecules on the skin that originated from personal care products or from the host. One ingredient that lasts on the skin is propylene glycol, which is commonly used in deodorants and antiperspirants and added in relatively large amounts as a humectant to create a soft and sleek consistency [51]. As shown, daily use of personal care products is leading to high levels of exposure to these polymers. Such polymers cause contact dermatitis in a subset of the population [51, 52]. Our data reveal a lasting accumulation of these compounds on the skin, suggesting that it may be possible to reduce their dose in deodorants or frequency of application and consequently decrease the degree of exposure to such compounds. Formulation design of personal care products may be influenced by performing detailed outcome studies. In addition, longer term impact studies are needed, perhaps in multiple year follow-up studies, to assess if the changes we observed are permanent or if they will recover to the original state.

Some of the host- and microbiome-modified molecules were also detected consistently, such as acylcarnitines, bile acids, and certain steroids. This means that a portion of the molecular composition of a person’s skin is not influenced by the beauty products applied to the skin, perhaps reflecting the level of exercise for acylcarnitines [53, 54] or the liver (dominant location where they are made) or gallbladder (where they are stored) function for bile acids. The bile acid levels are not related to sex and do not change in amount during the course of this study. While bile acids are typically associated with the human gut microbiome [34, 55–58], it is unclear what their role is on the skin and how they get there. One hypothesis is that they are present in the sweat that is excreted through the skin, as this is the case for several food-derived molecules such as caffeine or drugs and medications that have been previously reported on the human skin [19] or that microbes synthesize them de novo [55]. The only reports we could find on bile acids being associated with the skin describe cholestasis and pruritus diseases. Cholestasis and pruritus in hepatobiliary disease have symptoms of skin bile acid accumulation that are thought to be responsible for severe skin itching [59, 60]. However, since bile acids were found in over 50% of the healthy volunteers, their detection on the skin is likely a common phenotype among the general population and not only reflective of disease, consistent with recent reports challenging these molecules as biomarkers of disease [59]. Other molecules that were detected consistently came from personal care products.

Aside from molecules that are person-specific and those that do not vary, there are others that can be modified via personal care routines. Most striking is how the personal care routines influenced changes in hormones and pheromones in a personalized manner. This suggests that there may be personalized recipes that make it possible to make someone more or less attractive to others via adjustments of hormonal and pheromonal levels through alterations in skin care.

## Conclusion

Here, we describe the utilization of an approach that combines metabolomics and microbiome analysis to assess the effect of modifying personal care regime on skin chemistry and microbes. The key findings are as follows: (1) Compounds from beauty products last on the skin for weeks after their first use despite daily showering. (2) Beauty products alter molecular and bacterial diversity as well as the dynamic and structure of molecules and bacteria on the skin. (3) Molecular and bacterial temporal variability is product-, site-, and person-specific, and changes are observed starting the first week of beauty product use. This study provides a framework for future investigations to understand how lifestyle characteristics such as diet, outdoor activities, exercise, and medications shape the molecular and microbial composition of the skin. These factors have been studied far more in their impact on the gut microbiome and chemistry than in the skin. Revealing how such factors can affect skin microbes and their associated metabolites may be essential to define long-term skin health by restoring the appropriate microbes particularly in the context of skin aging [61] and skin diseases [49] as has shown to be necessary for amphibian health [62, 63], or perhaps even create a precision skin care approach that utilizes the proper care ingredients based on the microbial and chemical signatures that could act as key players in host defense [49, 64, 65].

## Methods

### Subject recruitment and sample collection

Twelve individuals between 25 and 40 years old were recruited to participate in this study, six females and six males. Female volunteer 8 dropped out of the study as she developed a skin irritation during the T1–T3 phase. All volunteers signed a written informed consent in accordance with the sampling procedure approved by the UCSD Institutional Review Board (Approval Number 161730). Volunteers were required to follow specific instructions during 9 weeks. They were asked to bring in samples of their personal care products they used prior to T0 so they could be sampled as well. Following the initial timepoint time 0 and during the first 3 weeks (week 1–week 3), volunteers were asked not to use any beauty products (Fig. 1b). During the next 3 weeks (week 4–week 6), four selected commercial beauty products provided to all volunteers were applied once a day at specific body part (deodorant for the armpits, soothing foot powder between the toes, sunscreen for the face, and moisturizer for front forearms) (Fig. 1b, Additional file 3: Table S2 Ingredient list of beauty products). During the first 6 weeks, volunteers were asked to shower with a head to toe shampoo. During the last 3 weeks (week 7–week 9), all volunteers went back to their normal routine and used the personal care products used before the beginning of the study (Fig. 1b). Volunteers were asked not to shower the day before sampling. Samples were collected by the same three researchers to ensure consistency in sampling and the area sampled. Researchers examined every subject together and collected metabolomics and microbiome samples from each location together. Samples were collected once a week (from day 0 to day 68—10 timepoints total) for volunteers 1, 2, 3, 4, 5, 6, 7, 9, 10, 11, and 12, and on day 0 and day 6 for volunteer 8. For individuals 4, 9, and 10, samples were collected twice a week. Samples collected for 11 volunteers during 10 timepoints: 11 volunteers × 10 timepoints × 4 samples × 4 body sites = 1760. Samples collected from 3 selected volunteers during 9 additional timepoints: 3 volunteers × 9 timepoints × 4 samples × 4 body sites = 432. All samples were collected following the same protocol described in [18]. Briefly, samples were collected over an area of 2 × 2 cm, using pre-moistened swabs in 50:50 ethanol/water solution for metabolomics analysis or in Tris-EDTA buffer for 16S rRNA sequencing. Four samples were collected from each body part right and left side. The locations sampled were the face—upper cheek bone and lower jaw, armpit—upper and lower area, arm—front of the elbow (antecubitis) and forearm (antebrachium), and feet—in between the first and second toe and third and fourth toe. Including personal care product references, a total of 2275 samples were collected over 9 weeks and were submitted to both metabolomics and microbial inventories.

### Metabolite extraction and UPLC-Q-TOF mass spectrometry analysis

Skin swabs were extracted and analyzed using a previously validated workflow described in [18, 19]. All samples were extracted in 200 μl of 50:50 ethanol/water solution for 2 h on ice then overnight at − 20 °C. Swab sample extractions were dried down in a centrifugal evaporator then resuspended by vortexing and sonication in a 100 μl 50:50 ethanol/water solution containing two internal standards (fluconazole 1 μM and amitriptyline 1 μM). The ethanol/water extracts were then analyzed using a previously validated UPLC-MS/MS method [18, 19]. We used a ThermoScientific UltiMate 3000 UPLC system for liquid chromatography and a Maxis Q-TOF (Quadrupole-Time-of-Flight) mass spectrometer (Bruker Daltonics), controlled by the Otof Control and Hystar software packages (Bruker Daltonics) and equipped with ESI source. UPLC conditions of analysis are 1.7 μm C18 (50 × 2.1 mm) UHPLC Column (Phenomenex), column temperature 40 °C, flow rate 0.5 ml/min, mobile phase A 98% water/2% acetonitrile/0.1% formic acid (*v*/*v*), mobile phase B 98% acetonitrile/2% water/0.1% formic acid (*v*/*v*). A linear gradient was used for the chromatographic separation: 0–2 min 0–20% B, 2–8 min 20–99% B, 8–9 min 99–99% B, 9–10 min 0% B. Full-scan MS spectra (*m/z* 80–2000) were acquired in a data-dependant positive ion mode. Instrument parameters were set as follows: nebulizer gas (nitrogen) pressure 2 Bar, capillary voltage 4500 V, ion source temperature 180 °C, dry gas flow 9 l/min, and spectra rate acquisition 10 spectra/s. MS/MS fragmentation of 10 most intense selected ions per spectrum was performed using ramped collision induced dissociation energy, ranged from 10 to 50 eV to get diverse fragmentation patterns. MS/MS active exclusion was set after 4 spectra and released after 30 s.

Mass spectrometry data collected from the skin of 12 individuals can be found here MSV000081582.

### LC-MS data processing

LC-MS raw data files were converted to mzXML format using Compass Data analysis software (Bruker Daltonics). MS1 features were selected for all LC-MS datasets collected from the skin of 12 individuals and blank samples (total 2275) using the open-source software MZmine [66]—see Additional file 4: Table S3 for parameters. Subsequent blank filtering, total ion current, and internal standard normalization were performed (Additional file 5: Table S4) for representation of relative abundance of molecular features (Fig. 2, Additional file 1: Figure S1), principal coordinate analysis (PCoA) (Fig. 4). For steroid compounds in Fig. 5a–d, bile acids (Additional file 1: Figure S5A-D), and acylcarnitines (Additional file 1: Figure S5E, F) compounds, crop filtering feature available in MZmine [66] was used to identify each feature separately in all LC-MS data collected from the skin of 12 individuals (see Additional file 4: Table S3 for crop filtering parameters and feature finding in Additional file 6: Table S5).

Heatmap in Fig. 2 was constructed from the bucket table generated from LC-MS1 features (Additional file 7: Table S6) and associated metadata (Additional file 8: Table S7) using the Calour command line available here: https://github.com/biocore/calour. Calour parameters were as follows: normalized read per sample 5000 and cluster feature minimum reads 50. Procrustes and Pearson correlation analyses in Additional file 1: Figures S10 and S11 were performed using the feature table in Additional file 9: Table S8, normalized using the probabilistic quotient normalization method [67].

### 16S rRNA amplicon sequencing

16S rRNA sequencing was performed following the Earth Microbiome Project protocols [68, 69], as described before [18]. Briefly, DNA was extracted using MoBio PowerMag Soil DNA Isolation Kit and the V4 region of the 16S rRNA gene was amplified using barcoded primers [70]. PCR was performed in triplicate for each sample, and V4 paired-end sequencing [70] was performed using Illumina HiSeq (La Jolla, CA). Raw sequence reads were demultiplexed and quality controlled using the defaults, as provided by QIIME 1.9.1 [71]. The primary OTU table was generated using Qiita (https://qiita.ucsd.edu/), using UCLUST (https://academic.oup.com/bioinformatics/article/26/19/2460/230188) closed-reference OTU picking method against GreenGenes 13.5 database [72]. Sequences can be found in EBI under accession number EBI: ERP104625 or in Qiita (qiita.ucsd.edu) under Study ID 10370. Resulting OTU tables were then rarefied to 10,000 sequences/sample for downstream analyses (Additional file 10 Table S9). See Additional file 11: Table S10 for read count per sample and Additional file 1: Figure S13 representing the samples that fall out with rarefaction at 10,000 threshold. The dataset includes 35 blank swab controls and 699 empty controls. The blank samples can be accessed through Qiita (qiita.ucsd.edu) as study ID 10370 and in EBI with accession number EBI: ERP104625. Blank samples can be found under the metadata category “sample_type” with the name “empty control” and “Swabblank.” These samples fell below the rarefaction threshold at 10,000 (Additional file 11: Table S10).

To rule out the possibility that personal care products themselves contained the microbes that induced the changes in the armpit and foot microbiomes that were observed in this study (Fig. 7), we subjected the common personal care products that were used in this study during T4–T6 also to 16S rRNA sequencing. The data revealed that within the limit of detectability of the current experiment, few 16S signatures were detected. One notable exception was the most dominant plant-originated bacteria chloroplast detected in the sunscreen lotion applied on the face (Additional file 1: Figure S9D), that was also detected on the face of individuals and at a lower level on their arms, sites where stable microbial communities were observed over time (Additional file 1: Figure S9E, F). This finding is in agreement with our previous data from the 3D cartographical skin maps that revealed the presence of co-localized chloroplast and lotion molecules [18]. Other low-abundant microbial signatures found in the sunscreen lotion include additional plant-associated bacteria: mitochondria [73], *Bacillaceae* [74, 75], *Planococcaceae* [76], and *Ruminococcaceae* family [77], but all these bacteria are not responsible for microbial changes associated to beauty product use, as they were poorly detected in the armpits and feet (Fig. 7).

To assess the origin of Cyanobacteria detected in skin samples, each Greengenes [72] 13_8 97% OTU table (per lane; obtained from Qiita [78] study 10,370) was filtered to only features with a p__Cyanobacteria phylum. The OTU maps for these tables—which relate each raw sequence to an OTU ID—were then filtered to only those observed p__Cyanobacteria OTU IDs. The filtered OTU map was used to extract the raw sequences into a single file. Separately, the unaligned Greengenes 13_8 99% representative sequences were filtered into two sets, first the set of representatives associated with c__Chloroplast (our interest database), and second the set of sequences associated with p__Cyanobacteria without the c__Chloroplast sequences (our background database). Platypus Conquistador [79] was then used to determine what reads were observed exclusively in the interest database and not in the background database. Of the 4,926,465 raw sequences associated with a p__Cyanobacteria classification (out of 318,686,615 total sequences), at the 95% sequence identity level with 100% alignment, 4,860,258 sequences exclusively recruit to full-length chloroplast 16S by BLAST [80] with the bulk recruiting to streptophytes (with Chlorophyta and Stramenopiles to a lesser extent). These sequences do not recruit non-chloroplast Cyanobacteria full length 16S.

### Half-life calculation for metabolomics data

In order to estimate the biological half-life of molecules detected in the skin, the first four timepoints of the study (T0, T1, T2, T3) were considered for the calculation to allow the monitoring of personal beauty products used at T0. The IUPAC’s definition of biological half-life as the time required to a substance in a biological system to be reduced to half of its value, assuming an approximately exponential removal [81] was used. The exponential removal can be described as *C*_(*t*)_ = *C*_0_*e*^−^^*tλ*^ where *t* represents the time in weeks, *C*_0_ represents the initial concentration of the molecule, *C*_(*t*)_ represents the concentration of the molecule at time *t*, and *λ* is the rate of removal [http://onlinelibrary.wiley.com/doi/10.1002/9780470140451.ch2/summary]. The parameter *λ* was estimated by a mixed linear effects model in order to account for the paired sample structure. The regression model tests the null hypothesis that *λ* is equal to zero and only the significant (*p* value < 0.05) parameters were considered.

### Principal coordinate analysis

We performed principal coordinate analysis (PCoA) on both metabolomics and microbiome data. For metabolomics, we used MS1 features (Additional file 5: Table S4) and calculated Bray–Curtis dissimilarity metric using ClusterApp (https://github.com/mwang87/q2_metabolomics).

For microbiome data, we used rarefied OTU table (Additional file 10: Table S9) and used unweighted UniFrac metric [36] to calculate beta diversity distance matrix using QIIME2 (https://qiime2.org). Results from both data sources were visualized using Emperor (https://biocore.github.io/emperor/) [28].

### Molecular networking

Molecular networking was generated from LC-MS/MS data collected from skin samples of 11 individuals MSV000081582, using the Global Natural Products Social Molecular Networking platform (GNPS) [29]. Molecular network parameters for MS/MS data collected from all body parts of 11 individuals during T0–T9 MSV000081582 are accessible here http://gnps.ucsd.edu/ProteoSAFe/status.jsp?task=284fc383e4c44c4db48912f01905f9c5. Molecular network parameters for MS/MS data collected from armpits T0–T3 MSV000081582 and deodorant used by individual 1 and 3 MSV000081580 can be found here http://gnps.ucsd.edu/ProteoSAFe/status.jsp?task=f5325c3b278a46b29e8860ec57915ad and here http://gnps.ucsd.edu/ProteoSAFe/status.jsp?task=aaa1af68099d4c1a87e9a09f398fe253, respectively. Molecular networks were exported and visualized in Cytoscape 3.4.0. [82]. Molecular networking parameters were set as follows: parent mass tolerance 1 Da, MS/MS fragment ion tolerance 0.5 Da, and cosine threshold 0.65 or greater, and only MS/MS spectral pairs with at least 4 matched fragment ions were included. Each MS/MS spectrum was only allowed to connect to its top 10 scoring matches, resulting in a maximum of 10 connections per node. The maximum size of connected components allowed in the network was 600, and the minimum number of spectra required in a cluster was 3. Venn diagrams were generated from Cytoscape data http://gnps.ucsd.edu/ProteoSAFe/status.jsp?task=284fc383e4c44c4db48912f01905f9c5 using Cytoscape [82] Venn diagram app available here http://apps.cytoscape.org/apps/all.

### Shannon molecular and bacterial diversity

The diversity analysis was performed separately for 16S rRNA data and LC-MS data. For each sample in each feature table (LC-MS data and microbiome data), we calculated the value of the Shannon diversity index. For LC-MS data, we used the full MZmine feature table (Additional file 5: Table S4). For microbiome data, we used the closed-reference BIOM table rarefied to 10,000 sequences/sample. For diversity changes between timepoints, we aggregated Shannon diversity values across groups of individuals (all, females, males) and calculated mean values and standard errors. All successfully processed samples (detected features in LC-MS or successful sequencing with 10,000 or more sequences/sample) were considered.

### Beauty products and chemical standards

Samples (10 mg) from personal care products used during T0 and T7–T9 MSV000081580 (Additional file 2: Table S1) and common beauty products used during T4–T6 MSV000081581 (Additional file 3: Table S2) were extracted in 1 ml 50:50 ethanol/water. Sample extractions were subjected to the same UPLC-Q-TOF MS method used to analyze skin samples and described above in the section “Metabolite extraction and UPLC-Q-TOF mass spectrometry analysis.” Authentic chemical standards MSV000081583 including 1-dehydroandrostenedion (5 μM), chenodeoxyglycocholic acid (5 μM), dehydroisoandrosterone sulfate (100 μM), glycocholic acid (5 μM), and taurocholic acid (5 μM) were analyzed using the same mass spectrometry workflow used to run skin and beauty product samples.

### Monitoring beauty product ingredients in skin samples

In order to monitor beauty product ingredients used during T4–T6, we selected only molecular features present in each beauty product sample (antiperspirant, facial lotion, body moisturizer, soothing powder) and then filtered the aligned MZmine feature table (Additional file 5: Table S4) for the specific feature in specific body part samples. After feature filtering, we selected all features that had a higher average intensity on beauty product phase (T4–T6) compared to non-beauty product phase (T1–T3). The selected features were annotated using GNPS dereplication output http://gnps.ucsd.edu/ProteoSAFe/status.jsp?task=69319caf219642a5a6748a3aba8914df, plotted using R package ggplot2 (https://cran.r-project.org/web/packages/ggplot2/index.html) and visually inspected for meaningful patterns.

### Random forest analysis

Random forest analysis was performed in MetaboAnalyst 3.0 online platform http://www.metaboanalyst.ca/faces/home.xhtml. Using LC-MS1 features found in armpit samples collected on T3 and T6. Random forest parameters were set as follows: top 1000 most abundant features, number of predictors to try for each node 7, estimate of error rate (0.0%).

### BugBase analysis

To determine the functional potential of microbial communities within our samples, we used BugBase [83]. Because we do not have direct access to all of the gene information due to the use of 16S rRNA marker gene sequencing, we can only rely on phylogenetic information inferred from OTUs. BugBase takes advantage of this information to predict microbial phenotypes by associating OTUs with gene content using PICRUSt [84]. Thus, using BugBase, we can predict such phenotypes as Gram staining, or oxidative stress tolerance at each timepoint or each phase. All statistical analyses in BugBase are performed using non-parametric differentiation tests (Mann–Whitney *U*).

### Taxonomic plots

Rarefied OTU counts were collapsed according to the OTU’s assigned family and genus name per sample, with a single exception for the class of chloroplasts. Relative abundances of each family-genus group are obtained by dividing by overall reads per sample, i.e., 10,000. Samples are grouped by volunteer, body site, and time/phase. Abundances are aggregated by taking the mean overall samples, and resulting abundances are again normalized to add up to 1. Low-abundant taxa are not listed in the legend and plotted in grayscale. Open-source code is available at https://github.com/sjanssen2/ggmap/blob/master/ggmap/snippets.py

### Dissimilarity-based analysis

Pairwise dissimilarity matrices were generated for metabolomics and 16S metagenomics quantification tables, described above, using Bray–Curtis dissimilarity through QIIME 1.9.1 [71]. Those distance matrices were used to perform Procrustes analysis (QIIME 1.9.1), and Mantel test (scikit-bio version 0.5.1) to measure the correlation between the metabolome and microbiome over time. The metabolomics dissimilarities were used to perform the PERMANOVA test to assess the significance of body part grouping. The PCoA and Procrustes plots were visualized in EMPeror. The dissimilarity matrices were also used to perform distance tests, comparing the distances within and between individuals and distances from time 0 to times 1, 2, and 3 using Wilcoxon rank-sum tests (SciPy version 0.19.1) [19].

### Statistical analysis for molecular and microbial data

Statistical analyses were performed in R and Python (R Core Team 2018). Monotonic relationships between two variables were tested using non-parametric Spearman correlation tests. The *p* values for correlation significance were subsequently corrected using Benjamini and Hochberg false discovery rate control method. The relationship between two groups was tested using non-parametric Wilcoxon rank-sum tests. The relationship between multiple groups was tested using non-parametric Kruskal–Wallis test. The significance level was set to 5%, unless otherwise mentioned, and all tests were performed as two-sided tests.

## Additional files

Additional file 1: **Figure S1.** Beauty products ingredients persist on skin of participants. **Figure S2.** Beauty product application impacts the molecular and bacterial diversity on skin of 11 individuals while the chemical diversity from personal beauty products used by males and females on T0 is similar. **Figure S3.** Longitudinal impact of ceasing and resuming the use of beauty products on the molecular composition of the skin over time. **Figure S4.** Molecular networking to highlight MS/MS spectra found in each body part. **Figure S5.** Longitudinal abundance of bile acids and acylcarnitines in skin samples. **Figure S6.** Characterization of steroids in armpits samples. **Figure S7.** Characterization of bile acids in armpit samples. **Figure S8.** Characterization of Acylcarnitine family members in skin samples. **Figure S9.** Beauty products applied at one body part might affect other areas of the body, while specific products determine stability versus variability of microflora at each body site. **Figure S10.** Representation of Gram-positive bacteria over time and the molecular features from the shampoo detected on feet. **Figure S11.** Procrustes analysis to correlate the skin microbiome and metabolome over time. **Figure S12.** Correlation between specific molecules and bacteria that change over time in armpits of individual 11. **Figure S13.** Representation of the number of samples that were removed (gray) and those retained (blue) after rarefaction at 10,000 threshold. (DOCX 1140 kb)

Additional file 2: **Table S1.** List of personal (T0 and T7–9) beauty products and their frequency of use. (XLSX 30 kb)

Additional file 3: **Table S2.** List of ingredients of common beauty products used during T4–T6. (PDF 207 kb)

Additional file 4: **Table S3.** Mzmine feature finding and crop filtering parameters. (XLSX 4 kb)

Additional file 5: **Table S4.** Feature table for statistical analysis with blank filtering and total ion current normalization. (CSV 150242 kb)

Additional file 6: **Table S5.** Feature table for individual feature abundance in armpits. (XLSX 379 kb)

Additional file 7: **Table S6.** Feature table for Calour analysis. (CSV 91651 kb)

Additional file 8: **Table S7.** Metadata for Calour analysis. (TXT 129 kb)

Additional file 9: **Table S8.** feature table with Probabilistic quotient normalization for molecular–microbial analysis. (ZIP 29557 kb)

Additional file 10: **Table S9.** OTU table rarefied to 10,000 sequences per sample. (BIOM 9493 kb)

Additional file 11: **Table S10.** 16S rRNA sequencing read counts per sample. (TSV 2949 kb)
