# Supplementary material for: The impact of skin care products on skin chemistry and microbiome dynamics
Source: BMC Biol. 2019 Jun 12;17:47. doi: 10.1186/s12915-019-0660-6 (PMC6560912; doi:10.1186/s12915-019-0660-6)
Supplement: Supplementary file 1 — Figure S1. Beauty products ingredients persist on skin of participants. Figure S2. Beauty product application impacts the molecular and bacterial diversity on skin of 11 individuals while the chemical diversity from personal beauty products used by males and females on T0 is similar. Figure S3. Longitudinal impact of ceasing and resuming the use of beauty products on the molecular composition of the skin over time. Figure S4. Molecular networking to highlight MS/MS spectra found in each body part. Figure S5. Longitudinal abundance of bile acids and acylcarnitines in skin samples. Figure S6. Characterization of steroids in armpits samples. Figure S7. Characterization of bile acids in armpit samples. Figure S8. Characterization of Acylcarnitine family members in skin samples. Figure S9. Beauty products applied at one body part might affect other areas of the body, while specific products determine stability versus variability of microflora at each body site. Figure S10. Representation of Gram-positive bacteria over time and the molecular features from the shampoo detected on feet. Figure S11. Procrustes analysis to correlate the skin microbiome and metabolome over time. Figure S12. Correlation between specific molecules and bacteria that change over time in armpits of individual 11. Figure S13. Representation of the number of samples that were removed (gray) and those retained (blue) after rarefaction at 10,000 threshold. (DOCX 1140 kb) [file 12915_2019_660_MOESM1_ESM.docx]

**Additional file 1, supplementary Figures S1-S13**

**Title:** The impact of skin care products on skin chemistry and microbiome dynamics.

**Authors:** Amina Bouslimani^1#^, Ricardo da Silva^1#^, Tomasz Kosciolek^2^, Stefan Janssen^2,3^, Chris Callewaert^2,4^, Amnon Amir^2^, Kathleen Dorrestein^1^, Alexey V. Melnik^1^, Livia S. Zaramela^2^**,** Ji-Nu Kim^2^, Gregory Humphrey^2^, Tara Schwartz^2^, Karenina Sanders^2^, Caitriona Brennan^2^, Tal Luzzatto-Knaan^1^, Gail Ackermann^2^, Daniel McDonald^2^, Karsten Zengler^2,5,6^, Rob Knight^2,5,6,7^, Pieter C Dorrestein^1,2,5,8^.

1. Collaborative Mass Spectrometry Innovation Center, Skaggs School of Pharmacy and Pharmaceutical Sciences.
2. Department of Pediatrics, University of California San Diego, La Jolla, CA 92037
3. Department for Pediatric Oncology, Hematology and Clinical Immunology, University Children's Hospital, Medical Faculty, Heinrich-Heine-University Düsseldorf.
4. Center for Microbial Ecology and Technology, Ghent University, 9000 Ghent, Belgium
5. Center for Microbiome Innovation, University of California, San Diego, La Jolla, CA 92307.
6. Department of Computer Science and Engineering, University of California, San Diego, La Jolla, CA 92093.
7. Department of Bioengineering, University of California, San Diego, La Jolla, CA 92037.
8. Department of Pharmacology, University of California, San Diego, La Jolla, CA 92037.

To whom correspondence should be addressed regarding microbial sequencing [rknight@ucsd.edu](mailto:rknight@ucsd.edu), mass spectrometry, sampling and the project itself [pdorrestein@ucsd.edu](mailto:pdorrestein@ucsd.edu)

# denotes equal contribution.

**
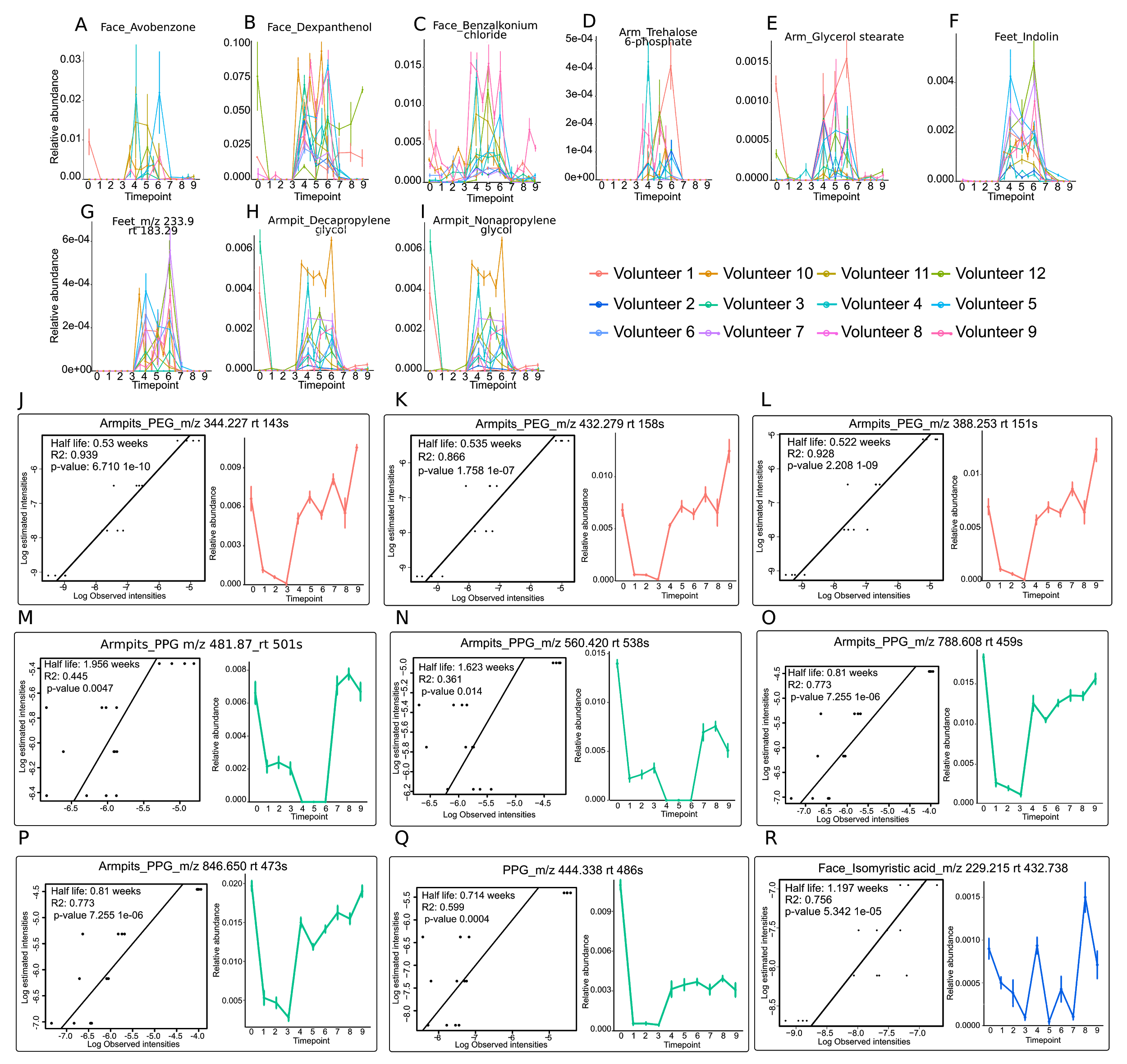
**

**Figure S1. Beauty products ingredients persist on skin of participants.** Ingredients from common beauty products used during T4-T6 were targeted in skin samples and their relative abundance at each body part was represented from T0 to T9. Ingredients from personal beauty products used before the experiment (T0) were also monitored over time and were observed when they went back to their original routine in T7-9. Error bars represent standard error of the mean calculated from four skin samples collected at each timepoint from right and left side of each body part of each individual separately.

(A-I) Plots represent ingredients from common beauty products used during T4-T6: sunscreen that was applied on face including A) Avobenzone, B) Dexpanthenol, C) Benzalkonium chloride, ingredients from moisturizer applied on arms D) Trehalose-6-phosphate, E) Glycerol stearate, compounds found in the foot powder F) Indolin, G) unannotated compound (m/z 233.9, rt 183.29) and ingredients from antiperspirant H) Decapropylene glycol and I) Nonapropylene glycol.

(J-L) Polyethylene glycol ingredients from personal deodorant used by volunteer 1 during T0 that persist in armpits up to 2 weeks after use.

(M-Q) Polypropylene glycol ingredients from personal deodorant used by volunteer 3 during T0 that persist in armpits up to 3 weeks after use.

(R) Myristic acid from face lotion used by volunteer 2 that persists on face, up to 2 weeks after use. Ingredients from personal face lotion used by volunteer 2 at T0 were monitored in skin samples and the relative abundance of myristic acid was represented from from T0 to T9.

See also Methods section for details about monitoring beauty products ingredients in skin samples.


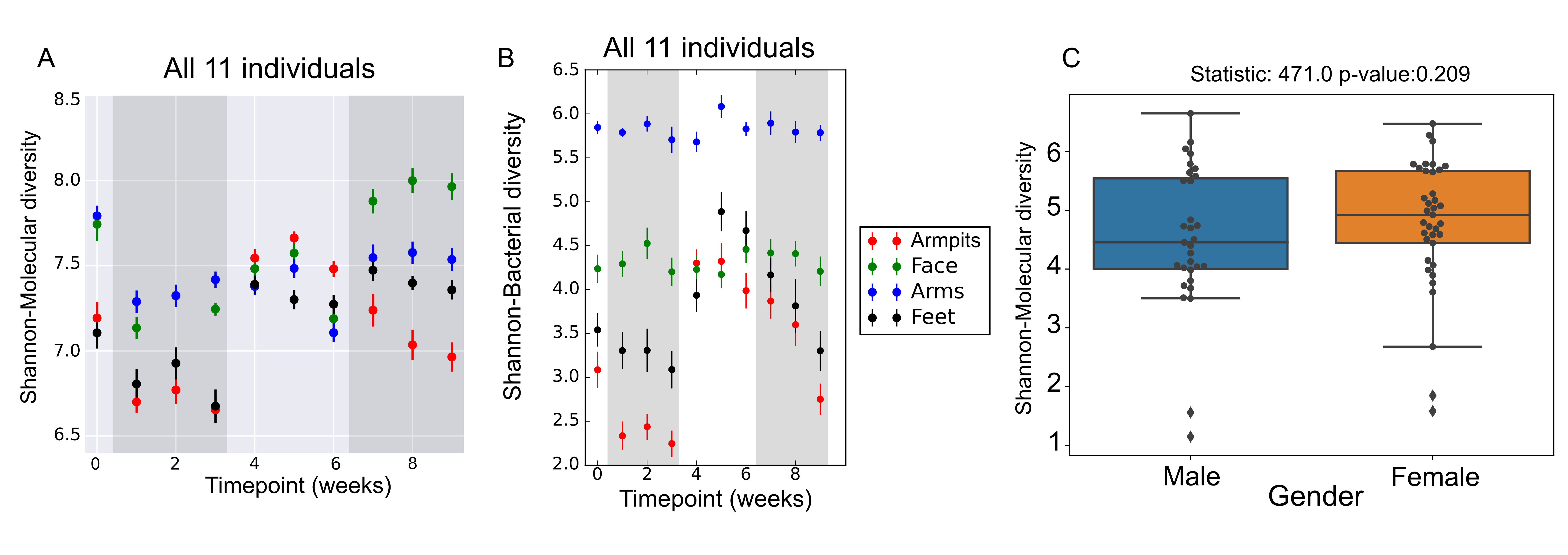


**Figure. S2. Beauty product application impacts the molecular and bacterial diversity on skin of 11 individuals while the chemical diversity from personal beauty products used by males and females on T0 is similar.**

(A, B) Molecular and bacterial diversity over a 9 week period, comparing A) metabolomics (UPLC-Q-TOF-MS) and B) microbial (16S rRNA sequencing) samples collected from all 11 individuals. Error bars represent standard error of the mean calculated at each timepoint from up to four samples collected from right and left side of each body part of 11 individuals.

(C) Molecular diversity using Shannon index comparing metabolomics data collected from personal beauty products used by females (30 samples, orange boxplot) and males (30 samples, blue boxplot) participants.


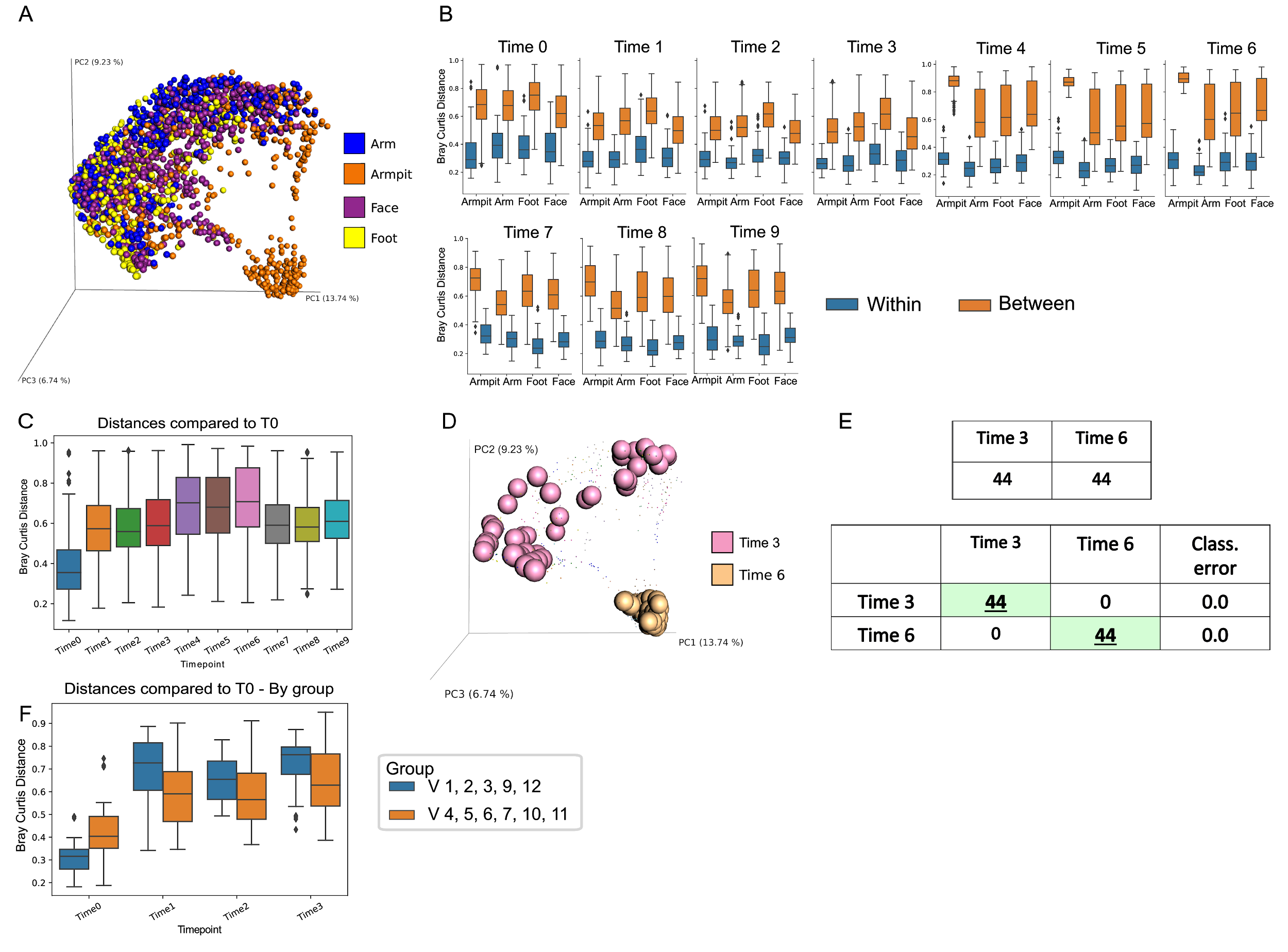


**Figure. S3. Longitudinal impact of ceasing and resuming the use of beauty products on the molecular composition of the skin over time.**

(A) Multivariate statistical analysis (Principal Coordinates Analysis PCoA) comparing mass spectrometry data collected from each body parts of 11 individuals. Color scale represents each body part: arm (blue), armpit (orange), foot (yellow), face (purple).

(B) Box-plot of Bray–Curtis distances over all metabolites were measured from subsets of collected LC-MS data to compare each body separately, within the same individual (blue) and between different individuals (orange).

(C) Box-plot of Bray-Curtis distances calculated between molecular profiles collected from skin of all 11 individuals, all body parts included, from T0 to T9.

(D) PCOA plot to visualize and compare, at the chemical level, molecular profiles collected from armpits of all individuals during Time 3 (no antiperspirant) and Time 6 (antiperspirant use). PCOA plots were generated using Bray Curtis matrix and visualized in emperor ([1](#_ENREF_1)).

(E) Random forest supervised classification analysis to predict molecular signatures from armpits collected at Time 3 and Time 6. Upper table: number of samples collected from armpits of individuals on Time 3 and Time 6. Lower table: Confusion matrix, output of random forest analysis. Green boxes with bold and underlined numbers represent the number of samples that were correctly predicted from each group. 100% of armpits samples from Time 3 and Time 6 were correctly predicted, suggesting unique molecular signatures that are associated to each phase. See also Supplementary Experimental Procedures for Random Forest parameters.

(F) Box-plot of Bray-Curtis distances calculated to compare skin samples collected from armpits of Group 1 (G1: Volunteers 1, 2, 3, 9, 12) and Group 2 (G2: volunteers 4, 5, 6, 7, 10, 11), during T0, T1, T2 and T3.


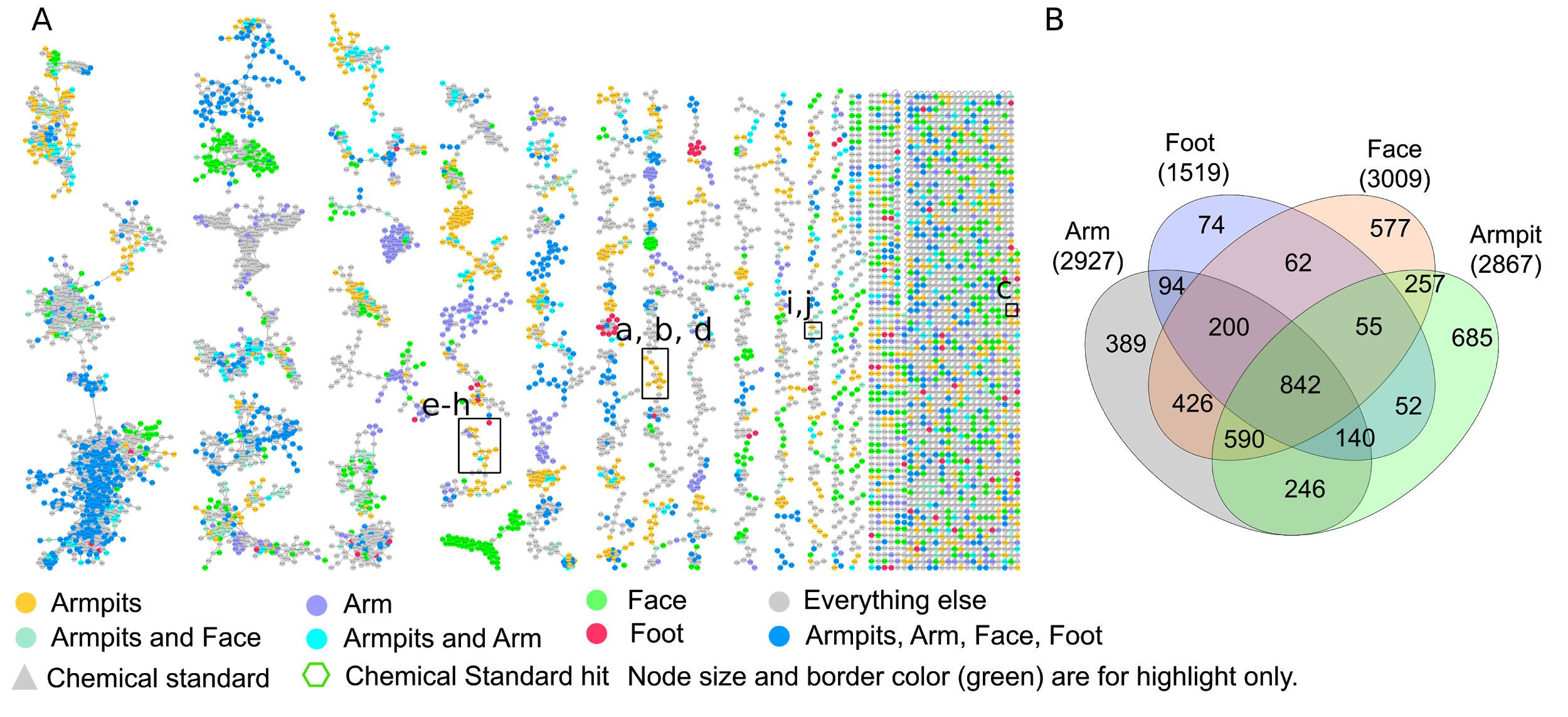


**Figure S4. Molecular networking to highlight MS/MS spectra found in each body part.**

(A) Full molecular network generated from LC-MS/MS data collected from skin of 11 healthy individuals [MSV000081582](http://massive.ucsd.edu/ProteoSAFe/dataset.jsp?task=148037acaf1d4679bb4b3835fb9acf06) and networking parameters can be found here <http://gnps.ucsd.edu/ProteoSAFe/status.jsp?task=284fc383e4c44c4db48912f01905f9c5>. Each node represents a consensus of a minimum of 3 MS/MS spectra. Each node color represents a specific body part or MS/MS spectra shared between 2 body parts. Hexagonal shape represents a hit to a chemical standard and triangle chemical standards. a-d), e-h) and i,j) represent molecular clusters shown in Figure 5 A-D, Figure S5A-D and Figure S5E, F, respectively.

(B) Venn diagram generated from LC-MC/MC clusters provided by the molecular network in A) and represents number of shared nodes (MS/MS spectra) between body parts (arm, foot, face, armpits) and nodes specific to each site.


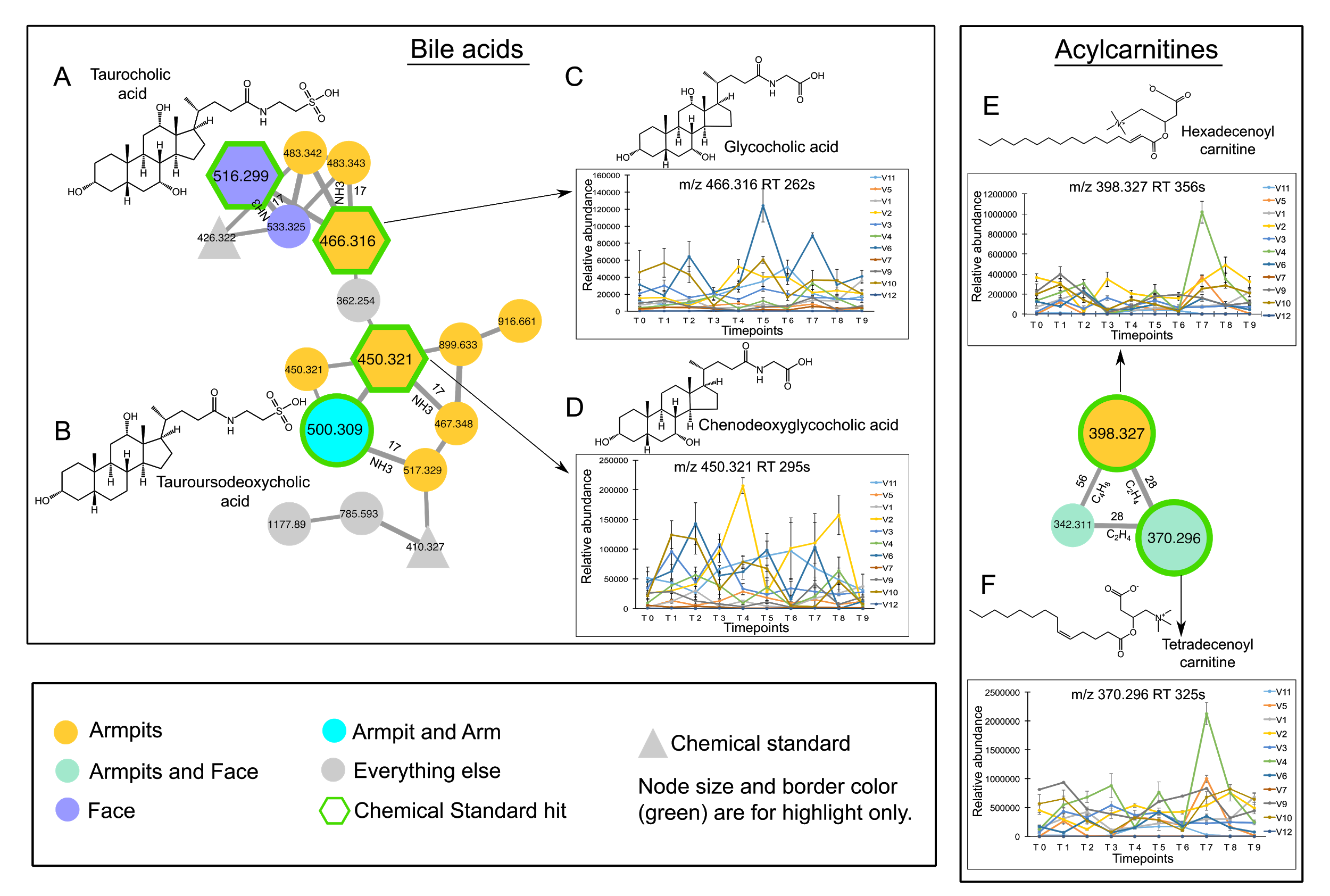


**Figure S5. Longitudinal abundance of bile acids and acylcarnitines in skin samples.** The full molecular network is shown in Figure. S4 and networking parameters can be found here <http://gnps.ucsd.edu/ProteoSAFe/status.jsp?task=284fc383e4c44c4db48912f01905f9c5> for MS/MS datasets [MSV000081582](http://massive.ucsd.edu/ProteoSAFe/dataset.jsp?task=148037acaf1d4679bb4b3835fb9acf06). Each node represents a consensus of a minimum of 3 identical MS/MS spectra. Each node color represents a specific body part or MS/MS spectra shared between 2 body parts. Hexagonal shape represents MS/MS spectra match between skin samples and chemical standards and triangles are chemical standards by themselves. Plots are representative of the relative abundance of each compound over time, calculated separately from LC-MS1 data collected from armpits of each individual.

(A-D) Bile acids molecular family including A) Taurocholic acid (*m/z* 516.299, rt 247s ), B) Tauroursodeoxycholic acid (*m/z* 500.309, rt 265s) C) Glycocholic acid (*m/z* 466.316, RT 262s) and D) Chenodeoxyglycocholic acid (*m/z* 450.321, rt 295s). Relative abundances are highlighted for Glycocholic acid and Chenodeoxyglycocholic acid.

(E-F) Acylcarnitine molecular family and relative abundance of 2 molecular species over a 9 weeks period. Relative abundances are represented for E) Hexadecenoyl carnitine (*m/z* 398,327, RT 356s) and F) Tetradecenoyl carnitine (*m/z* 370.296, RT 325s). See also Figures S6-S8 for the level of annotation of each compound. Error bars represent standard error of the mean calculated at each timepoint from four armpits samples collected from right and left side of each individual.

**
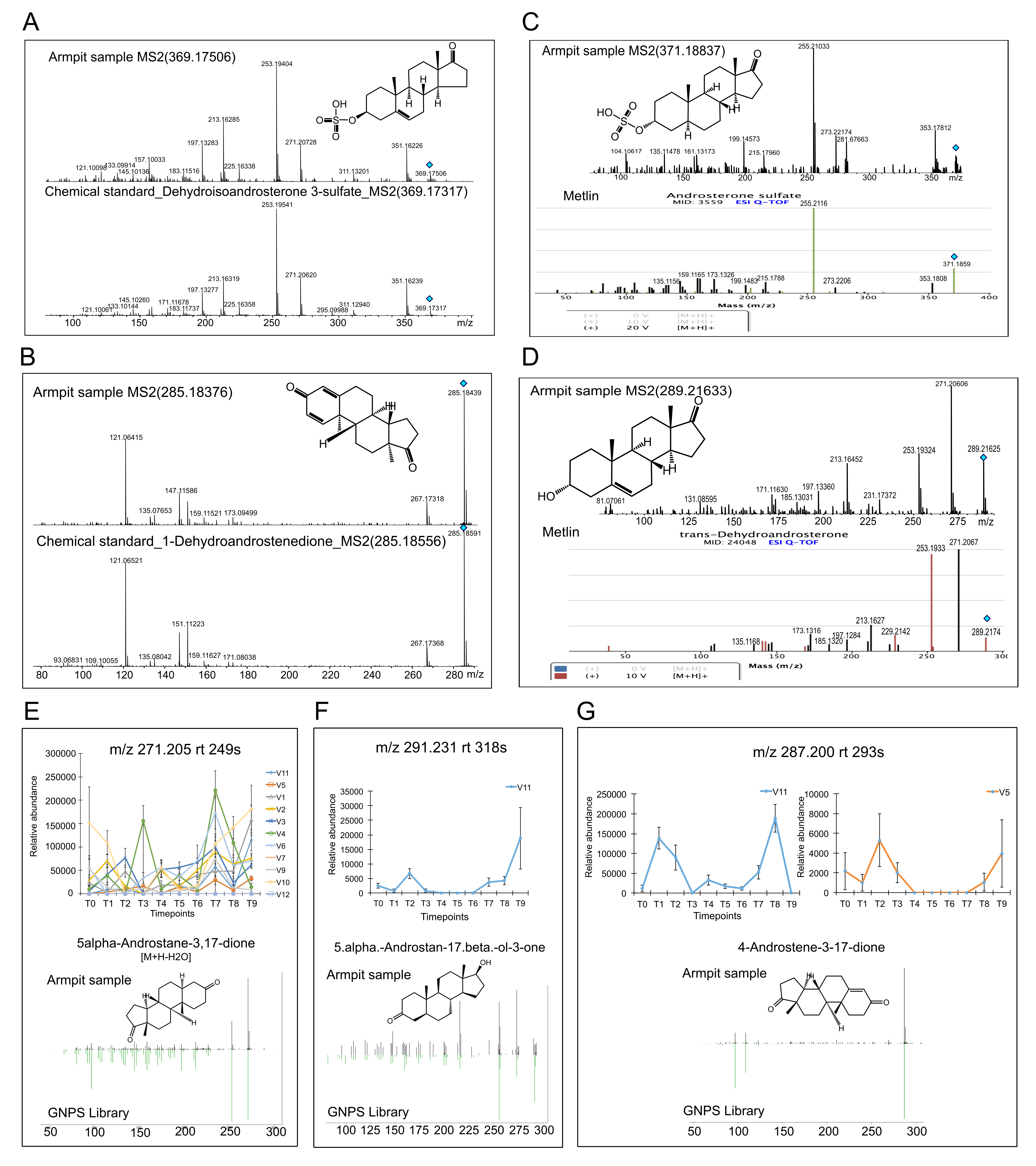
**

**Figure S6. Characterization of steroids in armpits samples.**

(A-D) Steroids including A) Dehydroisoandrosterone 3-sulfate (Armpit sample *m/z* 369.175, rt 250s - Standard *m/z* 369.173, rt 243s ), B) 1-Dehydroandrostenedione (Armpit sample *m/z* 285.184, rt 274s - Standard *m/z* 285.185, rt 271s) were validated using authentic standards based on MS/MS fragmentation and retention time. Note that retention time of Dehydroisoandrosterone 3-sulfate in armpit sample and chemical standard is slightly different consistent with positional isomers and therefore we classify as Level 3 ([2](#_ENREF_2)). 1-Dehydroandrostenedione was identified at a Level 1 based on MS/MS fragmentation and retention time match between armpit sample and authentic standard. C) Androsterone sulfate (*m/z* 371.188, rt 261s) and D) Dehydroandrosterone (*m/z* 289.216, rt 303s) were identified at a Level 3 based on MS/MS spectra matched between armpit sample and metlin library.

(E-G) represent temporal variation (upper panel) and match to GNPS library hit based on MS/MS fragmentation and precursor mass accuracy for compounds: E) 5alpha-Androstane-3.17-dione (Androstanedione) [M+H-H20] *m/z* 271.205 rt 249s, F) 5.alpha-Androstan-17.beta-ol-3-one (Androstanolone) [M+H]+ *m/z* 291.231 rt 318s and G) 4-Androstene-3-17-dione (Androstenedione) [M+H]+ *m/z* 287.200 rt 293s. Family members were annotated at a Level 3 ([2](#_ENREF_2)) based on MS/MS spectra match with GNPS library and precursor mass accuracy and molecular formula prediction. Error bars represent standard error of the mean calculated at each timepoint from four armpits samples collected from right and left side of each individual separately.


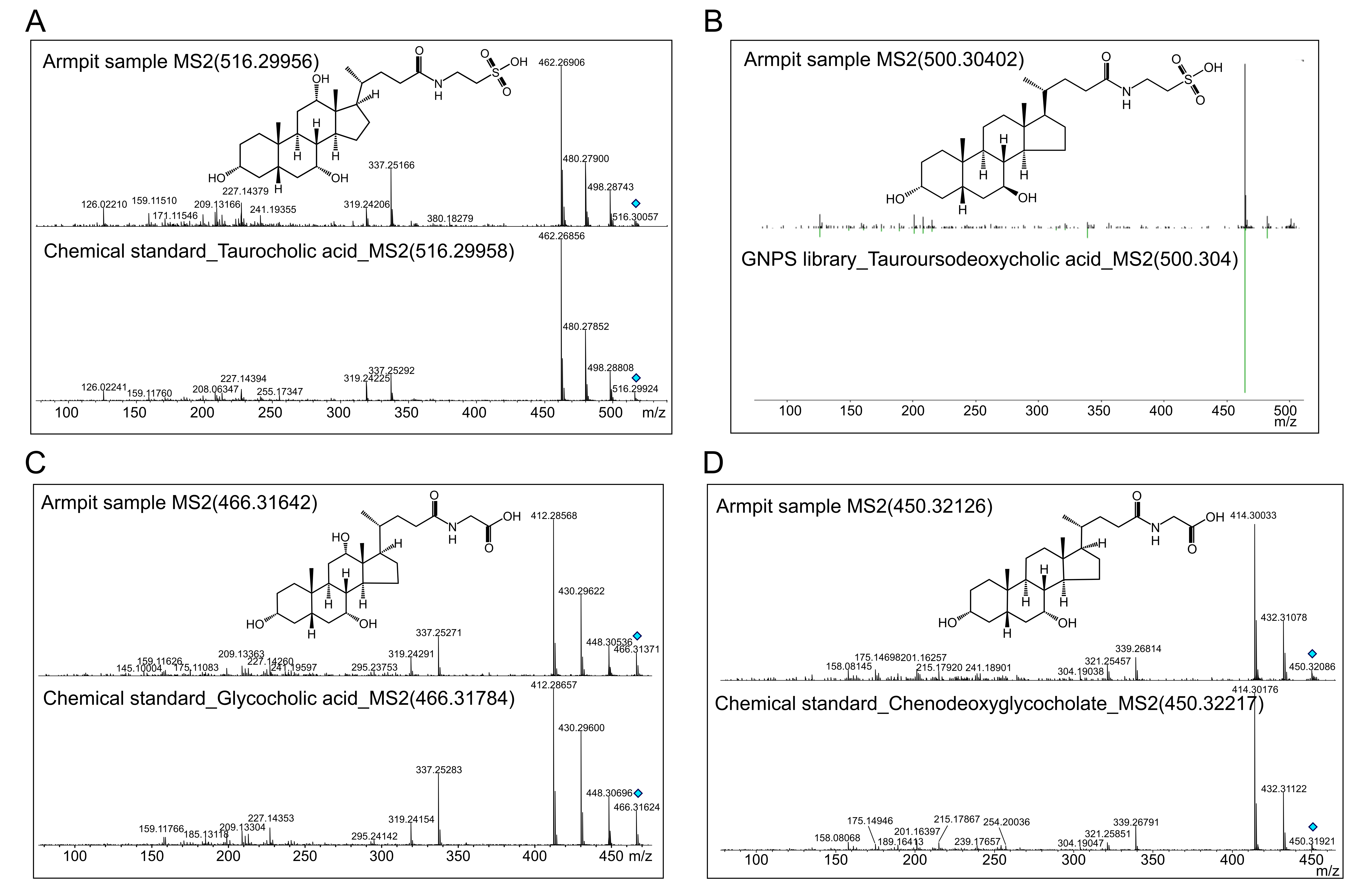


**Figure S7. Characterization of bile acids in armpit samples.**

(A) Tauraucholic acid (Armpit sample *m/z* 516.299, rt 247s - Standard *m/z* 516.299, rt 242s) identified at Level 1 using authentic standard, MS/MS and retention time match.

(B) Tauroursodeoxycholic (*m/z* 500.304, rt 265s) acid annotated at a Level 3 based on MS/MS match between armpit sample and GNPS library.

(C) Glycocholic acid (Armpit sample *m/z* 466.316, rt 263s - Standard *m/z* 466.317, rt 262s) identified at a Level 1 using authentic standard, MS/MS and retention time match.

(D) Chenodeoxyglycocholic acid (Armpit sample *m/z* 450.321, rt 294s *m/z* 450.322, rt 292s) identified at a Level 1 using authentic standard, MS/MS and retention time match.


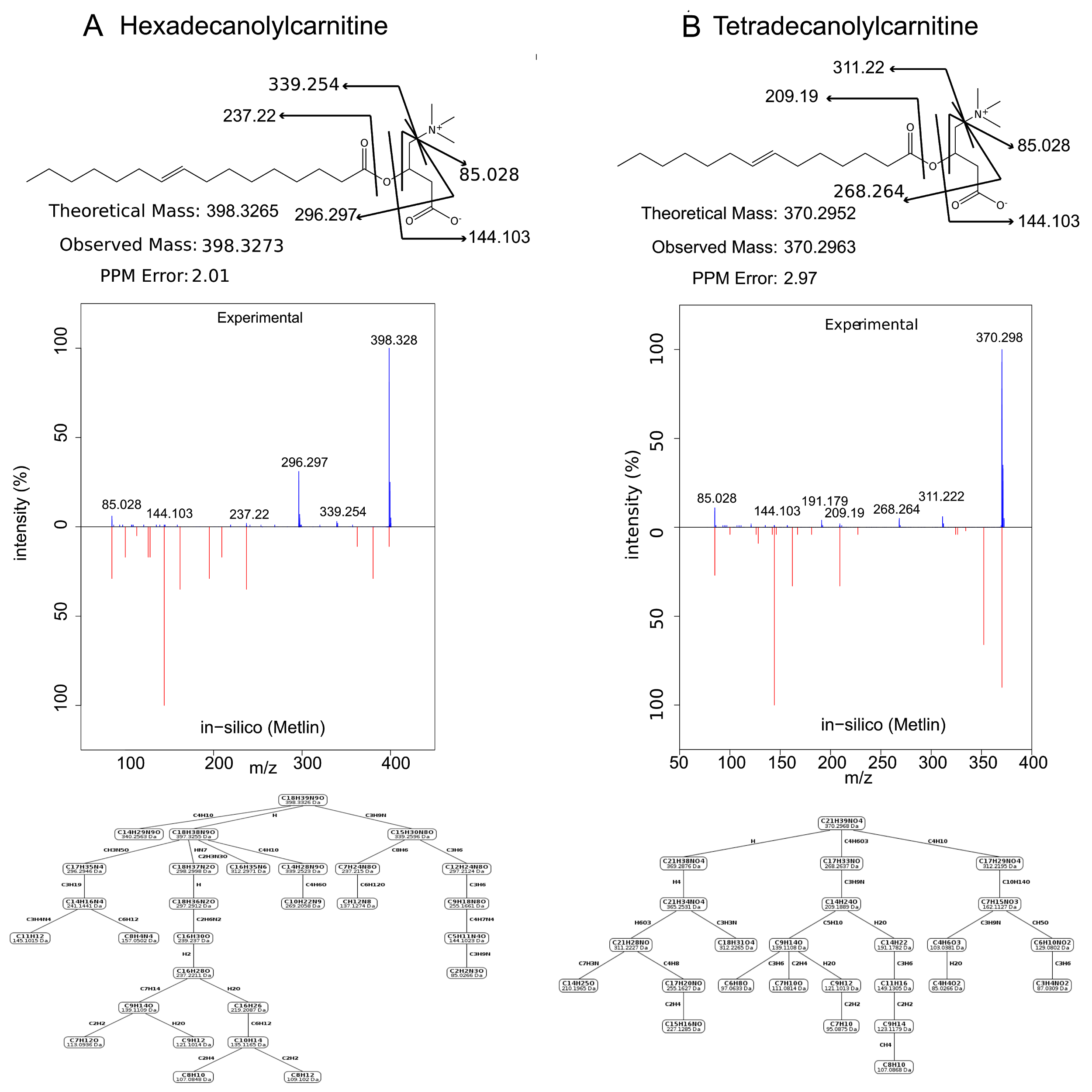


**Figure S8. Characterization of Acylcarnitine family members in skin samples.** Family members were annotated at a Level 3 ([2](#_ENREF_2)) based on MS/MS spectra match with Metlin library, precursor mass accuracy and molecular formula prediction.

(A) Annotation of Hexadecanolylcarnitine (*m/z* 398.3273, rt 356s) based on mass accuracy (upper panel), comparison of MS/MS fragmentation patterns from experimental skin sample and in-silico Metlin (middle panel) and prediction of molecular formula using Sirius CSI finger ID (lower panel).

(B) Annotation of Tetradecanoylcarnitine (*m/z* 370.2963, rt 325s) based on mass accuracy (upper panel), comparison of MS/MS spectra from experimental skin sample and in silico Metlin (middle panel) and prediction of molecular formula using Sirius CSI finger ID (lower panel).


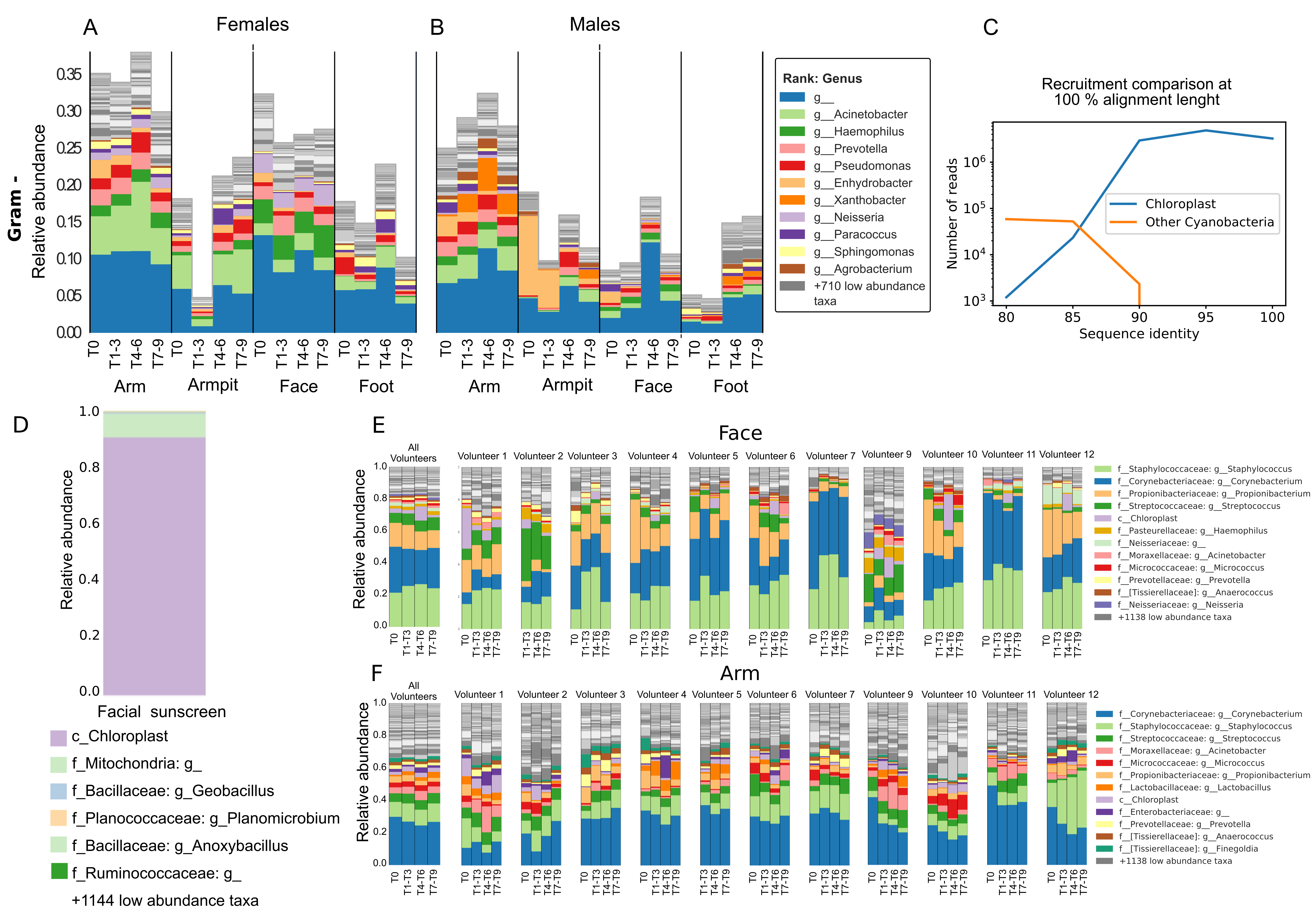


**Figure S9. Beauty products applied at one body part might affect other areas of the body, while specific products determine stability versus variability of microflora at each body site.**

(A, B) Gram - bacterial communities represented at the genus level from arm, armpit, face and foot of A) females and B) males over time.

(C) Platypus Conquistador ([3](#_ENREF_3)) database comparison of sequence reads classified as Cyanobacteria. Two databases were constructed from the Greengenes 13_8 99% OTUs ([4](#_ENREF_4)) one composed of Chloroplast full length 16S and one of non-Chloroplast Cyanobacteria. Through Platypus Conquistador, BLAST ([5](#_ENREF_5)) searches at increasing levels of sequence identity were performed against both databases. As the sequence identity threshold increases, there is a concomitant increase in reads with only recruit to the Chloroplast database. At all levels, zero reads recruiting to both databases.

(D) Representation of bacteria found in facial sunscreen lotion used during timepoints T4-T6. See also Methods section 16S rRNA Amplicon Sequencing.

(E, F) Stability of bacterial communities represented at the genus level from E) face and F) arm over time.


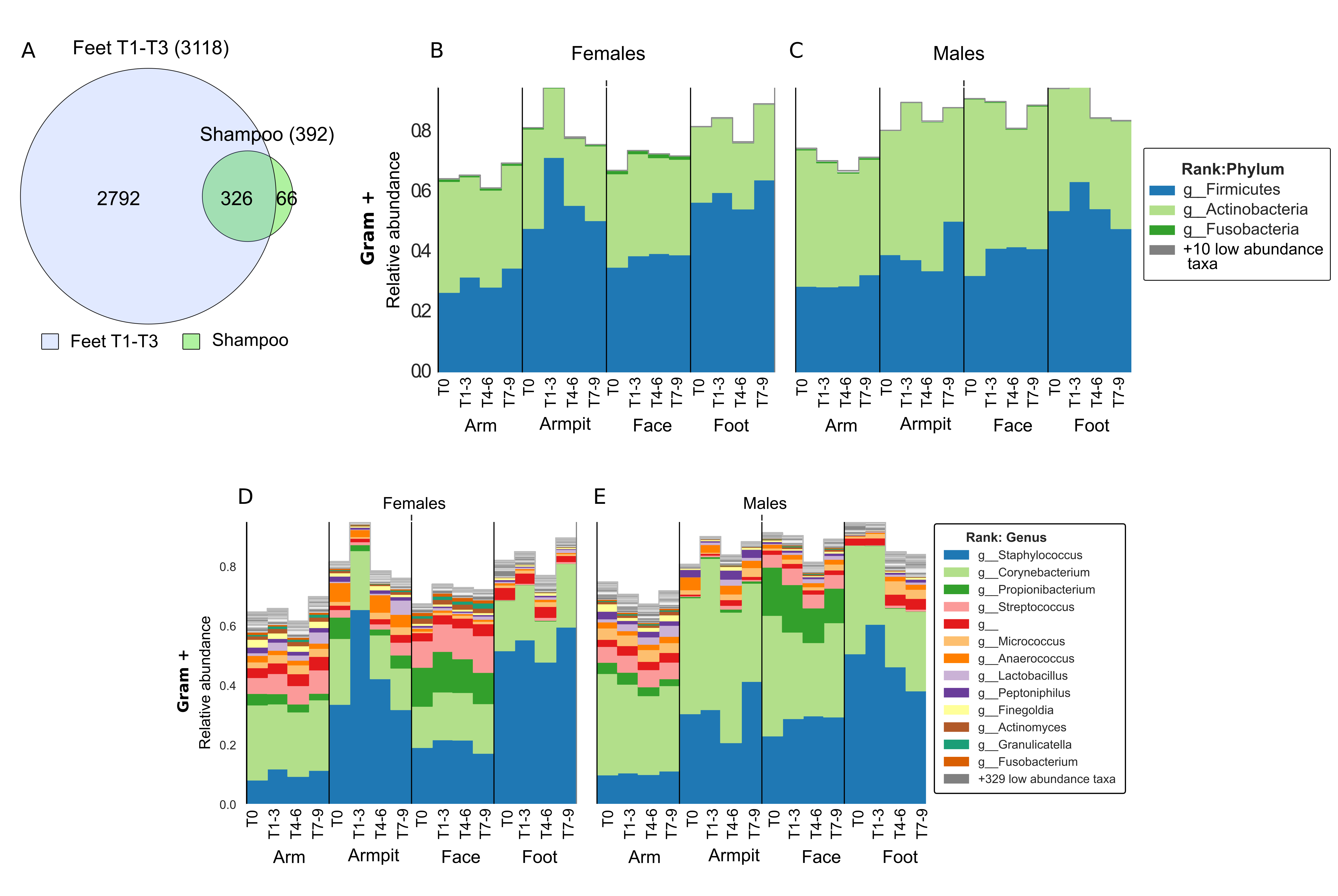


**Figure S10. Representation of Gram-positive bacteria over time and the molecular features from the shampoo detected on feet.**

(A) Chemical features found on feet samples and the shampoo. Venn diagram representing the number of nodes (326 out of 3184; ~10%) between Feet samples collected during T1-T3 and the head to toe shampoo used during T1-T, unique feet (2792 out of 3184; ~88%) and shampoo nodes (66 out of 3184; ~ 2%). Venn diagram was generated from molecular network <http://gnps.ucsd.edu/ProteoSAFe/status.jsp?task=540f765d826d414e938280e3441e52bc> generated between feet samples T1-T3 [MSV000081582](http://massive.ucsd.edu/ProteoSAFe/dataset.jsp?task=148037acaf1d4679bb4b3835fb9acf06) and the head to toe shampoo

| [MSV000081581](http://massive.ucsd.edu/ProteoSAFe/dataset.jsp?task=74edaecc4be44b21866bb30a6ebf0e92). |
| --- |

(B-E) Representation of Gram positive bacteria detected on each body site, at the B, C) phylum and D, E) genus level. Gram positive bacteria detected from armpits, feet, arms and face are represent for B, D) females and C, E) males. See Methods section BugBase analysis.


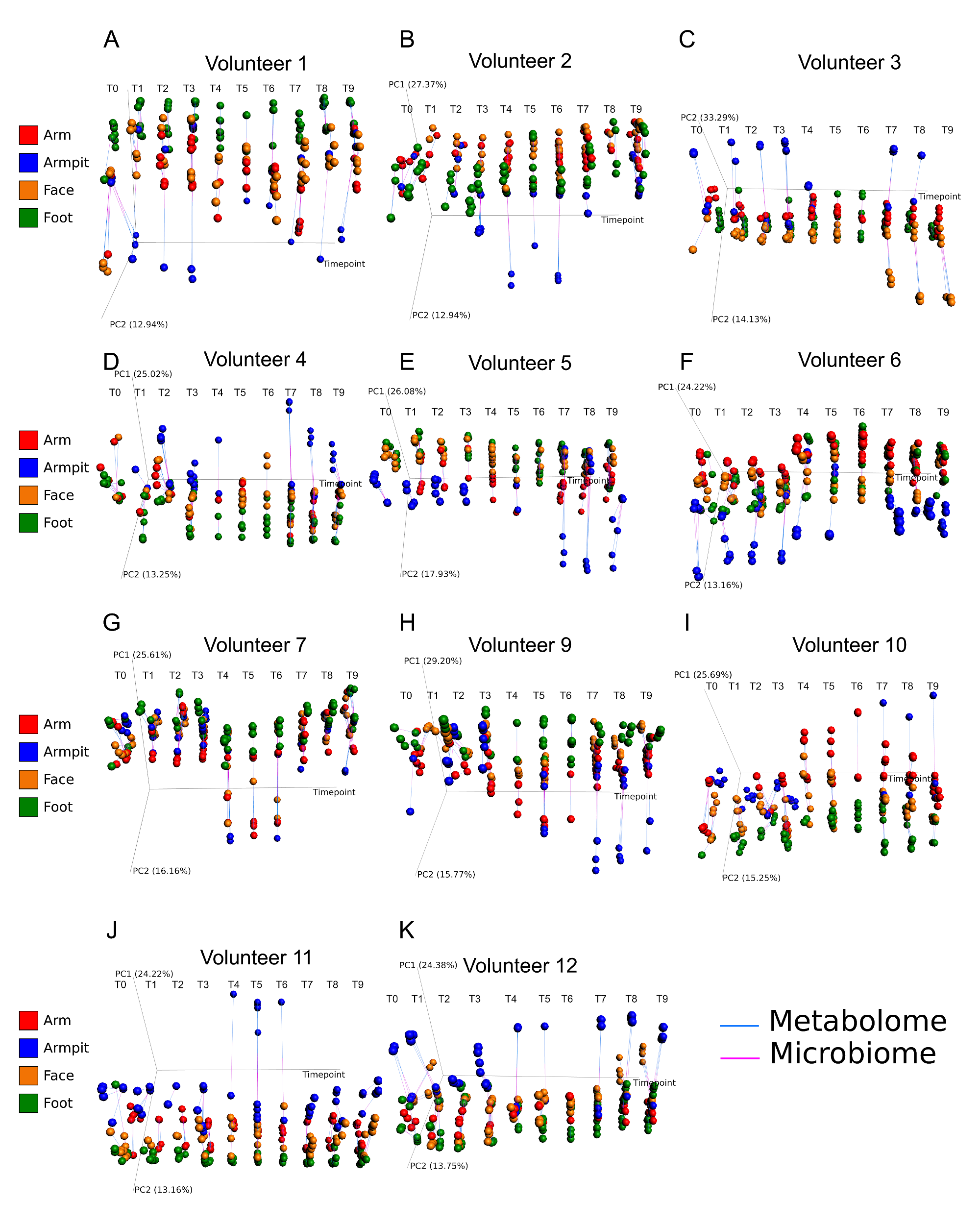


**Figure S11. Procrustes analysis to correlate the skin microbiome and metabolome over time.** Procrustes plots represent correlation of metabolomics and microbiome samples collected from on arm (red nodes), armpit (blue nodes), face (orange nodes) and foot (green nodes) separately for volunteers A) 1, B) 2, C) 3, D) 4, E) 5, F) 6, G) 7, H) 9, I) 10, J) 11 and K) 12, from T0 to T9. The coordinates for a sample obtained using metabolomics data (blue lines) are connected to coordinates for the same sample obtained using microbiome data (pink lines). The timepoint axis (in weeks) representing changes in hygiene routine from T0 to T9 is constrained to be one of the axes in the ordination plots. Note that samples (armpits samples from one or more timepoints during antiperspirant use) that had fewer than 10,000 reads were excluded from the analyses.


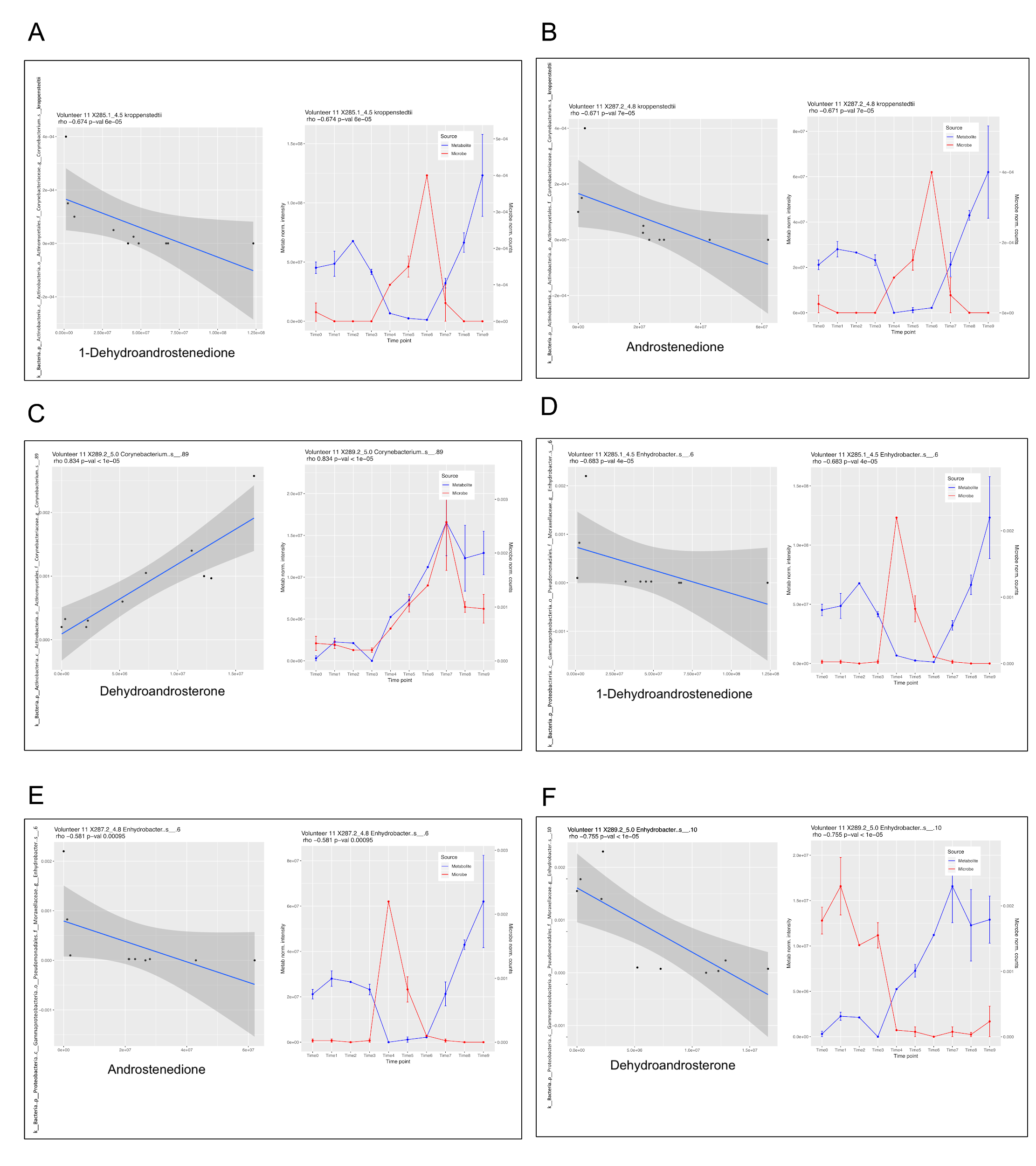


**Figure S12. Correlation between specific molecules and bacteria that change over time in armpits of individual 11.** Pearson correlation was used to associate molecular and bacterial patterns over time. Regression plots together with the metabolite and microbial pattern are presented. The pearson correlation coefficient and p values were calculated for each correlation and displayed on the top of each plot. Correlations include:

(A) 1-Dehydroandrostenedione and *Corynebacterium* genera - kroppenstedtii strain in armpits of volunteer 11 (r=-0.674, p=6e-05).

(B) Androstenedione and *Corynebacterium* genera - kroppenstedtii strain (r=-0.671, p=7e-05).

(C) Dehydroandrosterone and *Corynebacterium* genera - unknown strain number 89 (r=0.834, p<1e-05).

(D) 1-Dehydroandrostenedione and *Enhydrobacter* genera - unknown strain number 6 (r=-0.683, p=4e-05).

(E) Androstenedione and *Enhydrobacter* genera - unknown strain number 6 (r=-0.581, p=0.00095).

(F) Dehydroandrosterone and *Enhydrobacter* genera - unknown strain number 10 (r=-0.755, p<1e-05).

Left panel of A-F) corresponds to the regression plot and right panel molecular and bacterial relative abundance over time.


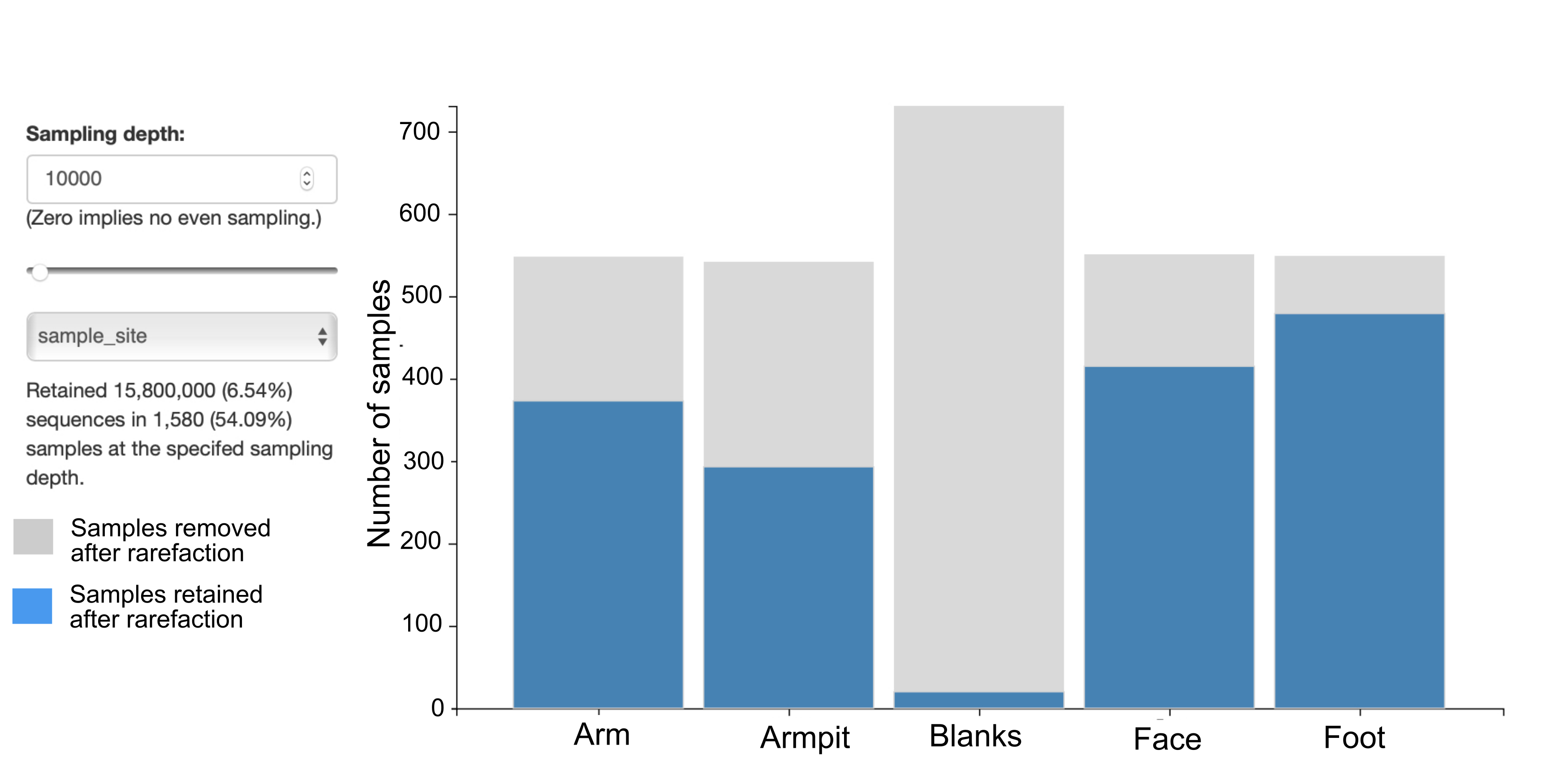


**Figure S13. Representation of the number of samples that were removed (grey) and those retained (blue) after rarefaction at 10,000 threshold.** The plot represents blank samples as well as skin samples from arm, armpit, face and foot collected from 11 individuals over 9 weeks.

**References.**

1. Vazquez-Baeza Y, Pirrung M, Gonzalez A, Knight R. EMPeror: a tool for visualizing high-throughput microbial community data. Gigascience. 2013 Nov 26;2(1):16.

2. Sumner LW, Amberg A, Barrett D, Beale MH, Beger R, Daykin CA, et al. Proposed minimum reporting standards for chemical analysis Chemical Analysis Working Group (CAWG) Metabolomics Standards Initiative (MSI). Metabolomics. 2007 Sep;3(3):211-21.

3. Gonzalez A, Vazquez-Baeza Y, Pettengill JB, Ottesen A, McDonald D, Knight R. Avoiding Pandemic Fears in the Subway and Conquering the Platypus. mSystems. 2016 May-Jun;1(3):e00050-16.

4. McDonald D, Price MN, Goodrich J, Nawrocki EP, DeSantis TZ, Probst A, et al. An improved Greengenes taxonomy with explicit ranks for ecological and evolutionary analyses of bacteria and archaea. ISME J. 2012 Mar;6(3):610-8.

5. Altschul SF, Gish W, Miller W, Myers EW, Lipman DJ. Basic local alignment search tool. J Mol Biol. 1990 Oct 5;215(3):403-10.
