# Supplementary material for: The impact of skin care products on skin chemistry and microbiome dynamics
Source: BMC Biol. 2019 Jun 12;17:47. doi: 10.1186/s12915-019-0660-6 (PMC6560912; doi:10.1186/s12915-019-0660-6)
Supplement: Supplementary file 3 — Table S2. List of ingredients of common beauty products used during T4–T6. (PDF 207 kb) [file 12915_2019_660_MOESM3_ESM.pdf]

<u>Ingredient list of beauty products used during T1-T6 and T4-T6.</u>

**Beauty product**	**Ingredients**	**Body part**	**Frequency of application**
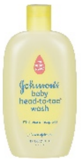 Johnson & Johnson Head-To-Toe Baby Wash	Water (eau), Cocamidopropyl Betaine, PEG-80 Sorbitan Laurate, Sodium Trideceth Sulfate, PEG-150 Distearate, Glycerin, Polyquaternium-10, Tetrasodium EDTA, Sodium Chloride, Citric Acid, Sodium Hydroxide, Sodium Benzoate, Ethylhexylglycerin, Phenoxyethanol, Parfum	Whole body	Daily, for 6 weeks (T 1-6)
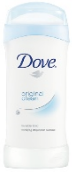 Dove Anti-Perspirant Deodorant, Original Clean Dove	Active Ingredient: Aluminum Zirconium Tetrachlorohydrex GLY (18.5%). Inactive Ingredients: Cyclopentasiloxane, Stearyl Alcohol, PPG-14 Butyl Ether, Dimethicone, C12-15 Alkyl Benzoate, Hydrogenated Castor Oil, Talc, Helianthus Annuus (Sunflower) Seed Oil, Fragrance, Steareth-100, Vegetable Oil, Glyceryl Oleate, Glycerin, Propylene Glycol, TBHQ, BHT, Citric Acid.	Armpits	Daily, for 3 weeks (T 4-6)
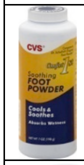 CVS/pharmacy Soothing Foot Powder	Talc, Salicylic Acid, Methyl Salicylate.	Foot	Daily, for 3 weeks (T 4-6)
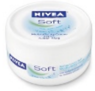 Nivea soft moisturizer cream	Water, Myristyl Alcohol, Glycerin, Mineral Oil, Butylene Glycol, Alcohol Denat., Stearic Acid, Petrolatum, Myristyl Myristate, Glyceryl Stearate, Hydrogenated Coco-Glycerides, Dimethicone, Simmondsia Chinensis (Jojoba) Seed Oil, Tocopheryl Acetate, Polyglyceryl-2 Caprate, Phenoxyethanol, Lanolin Alcohol, Fragrance, Carbomer, Sodium Hydroxide, Methylparaben, Ethylparaben, Propylparaben,	Arm	Daily, for 3 weeks (T 4-6)

	Butylparaben, Isobutylparaben.		
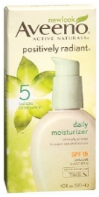 AVEENO® POSITIVELY RADIANT® DAILY MOISTURIZER BROAD SPECTRUM SPF 15	Active: Avobenzone (3%), Octinoxate (7.5%), Octisalate (2%) Purpose: Sunscreen Inactive: Water, C12-15 Alkyl Benzoate, Cetearyl Alcohol, Dimethicone, Glycine Soja (Soybean) Seed Extract, Glycerin, Bis-Phenylpropyl Dimethicone, Arachidyl Alcohol, Phenoxyethanol, Cetearyl Glucoside, Panthenol, Benzyl Alcohol, Ethylene/Acrylic Acid Copolymer, Behenyl Alcohol, Steareth-2, Fragrance, Steareth-21, Polyacrylamide, Polymethyl Methacrylate, Arachidyl Glucoside, Disodium EDTA, Methylparaben, C13-14 Isoparaffin, Ethylparaben, Butylparaben, Laureth-7, Benzalkonium Chloride , Propylparaben, Isobutylparaben, Iodopropynyl Butylcarbamate, BHT, Titanium Dioxide, Mica, Silica	Face	Daily, for 3 weeks (T 4-6)
